# Supplementary figures and images for: A macroecological perspective on genetic diversity in the human gut microbiome
Source: PLoS One. 2023 Jul 21;18(7):e0288926. doi: 10.1371/journal.pone.0288926 (PMC10361512; doi:10.1371/journal.pone.0288926)

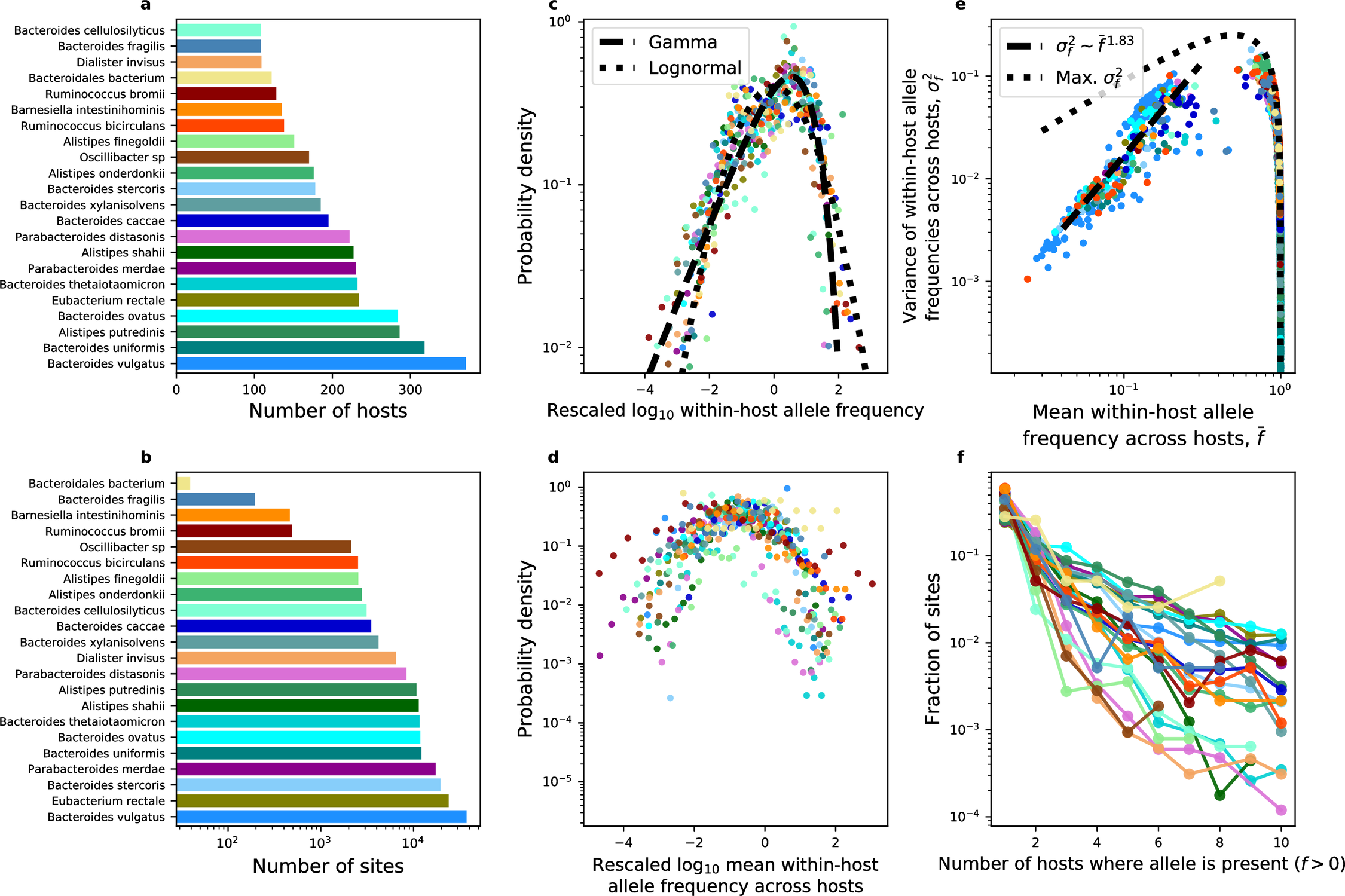

Supplement: S1 Fig — Measures of genetic diversity calculated from nonsynonymous sites exhibit similar statistical forms across phylogenetically distant species in the human gut, similar to patterns observed among synonymous sites (Fig 1). (TIF) [file pone.0288926.s001.tif]

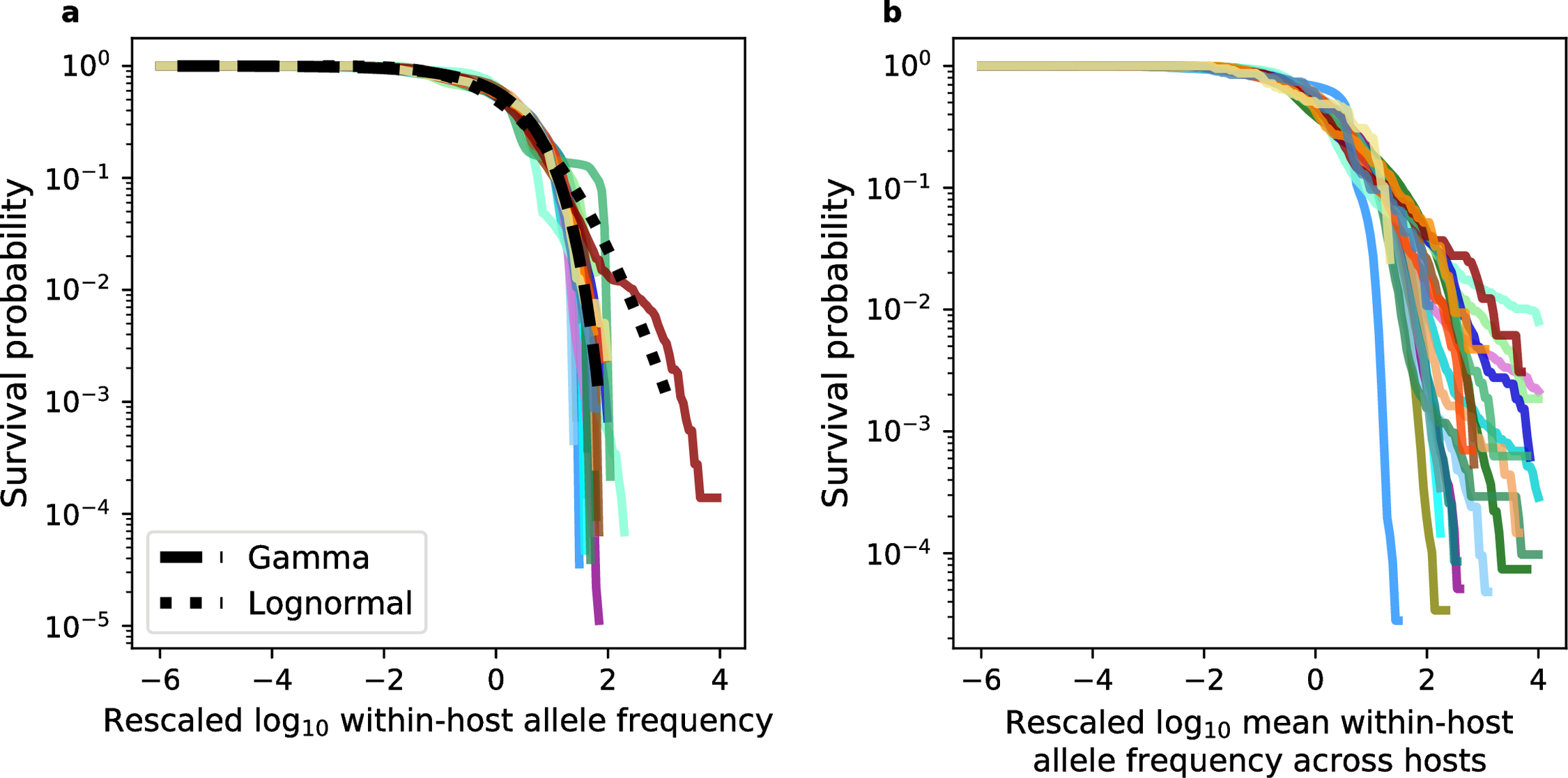

Supplement: S2 Fig — Survival forms of rescaled distributions of within-host allele frequencies across hosts and mean frequencies across hosts. Representing the data presented in Fig 1c and 1d reveals how distributions of genetic diversity have similar forms across phylogenetically distant species. Each non-black line represents a species. A dashed black line represents the fit of a gamma distribution and dotted black line represents a lognormal. (TIF) [file pone.0288926.s002.tif]

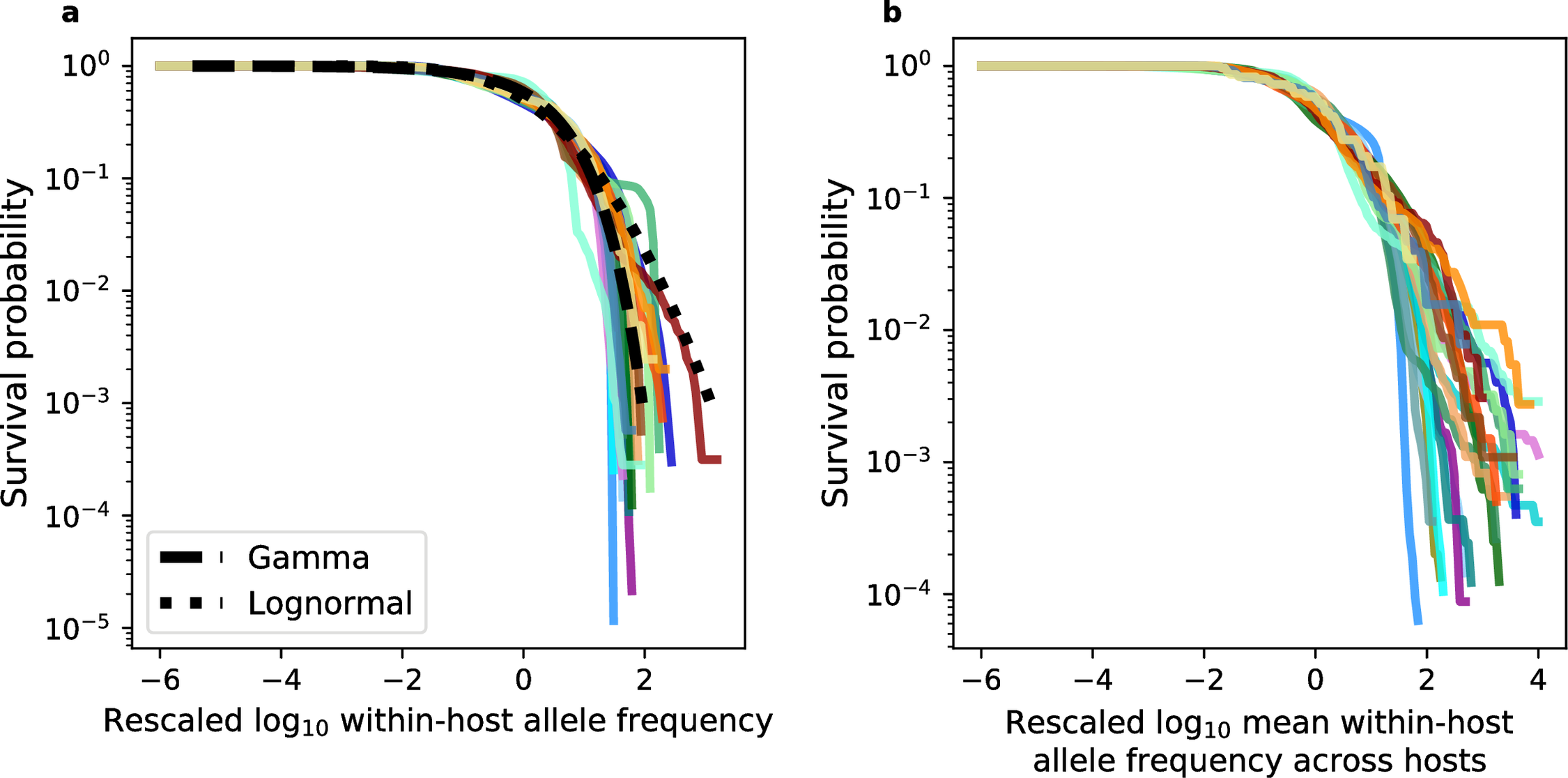

Supplement: S3 Fig — The equivalent plot for S2 Fig for nonsynonymous sites. (TIF) [file pone.0288926.s003.tif]

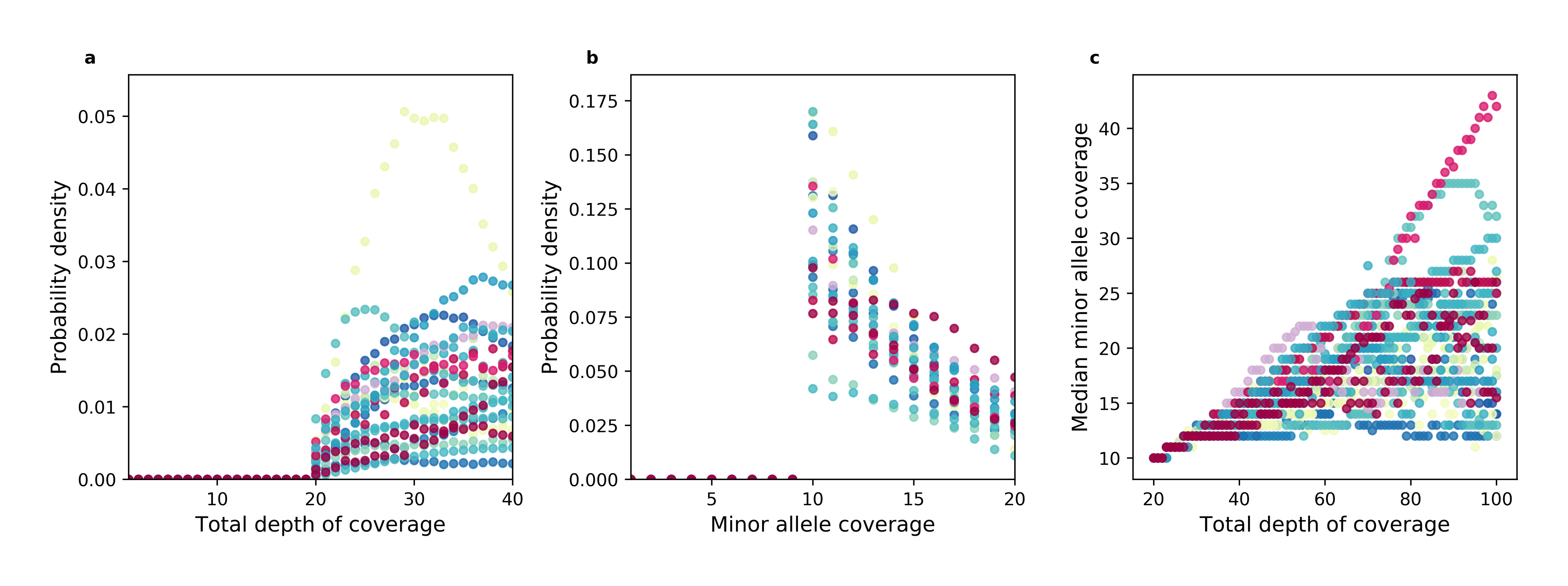

Supplement: S4 Fig — The use of the log-likelihood ratio in MAPGD introduces a lower bound on the total depth of coverage (D) necessary to estimate the frequency of an allele at a given site. a) The existence of a lower bound translates to a truncation of the data, where I did not observe any sites with a coverage less than 20 that were processed by MAPGD. b,c) This truncation means that the depth of coverage of a minor allele (A) cannot be less than half the total coverage (e.g., 10). (TIF) [file pone.0288926.s004.tif]

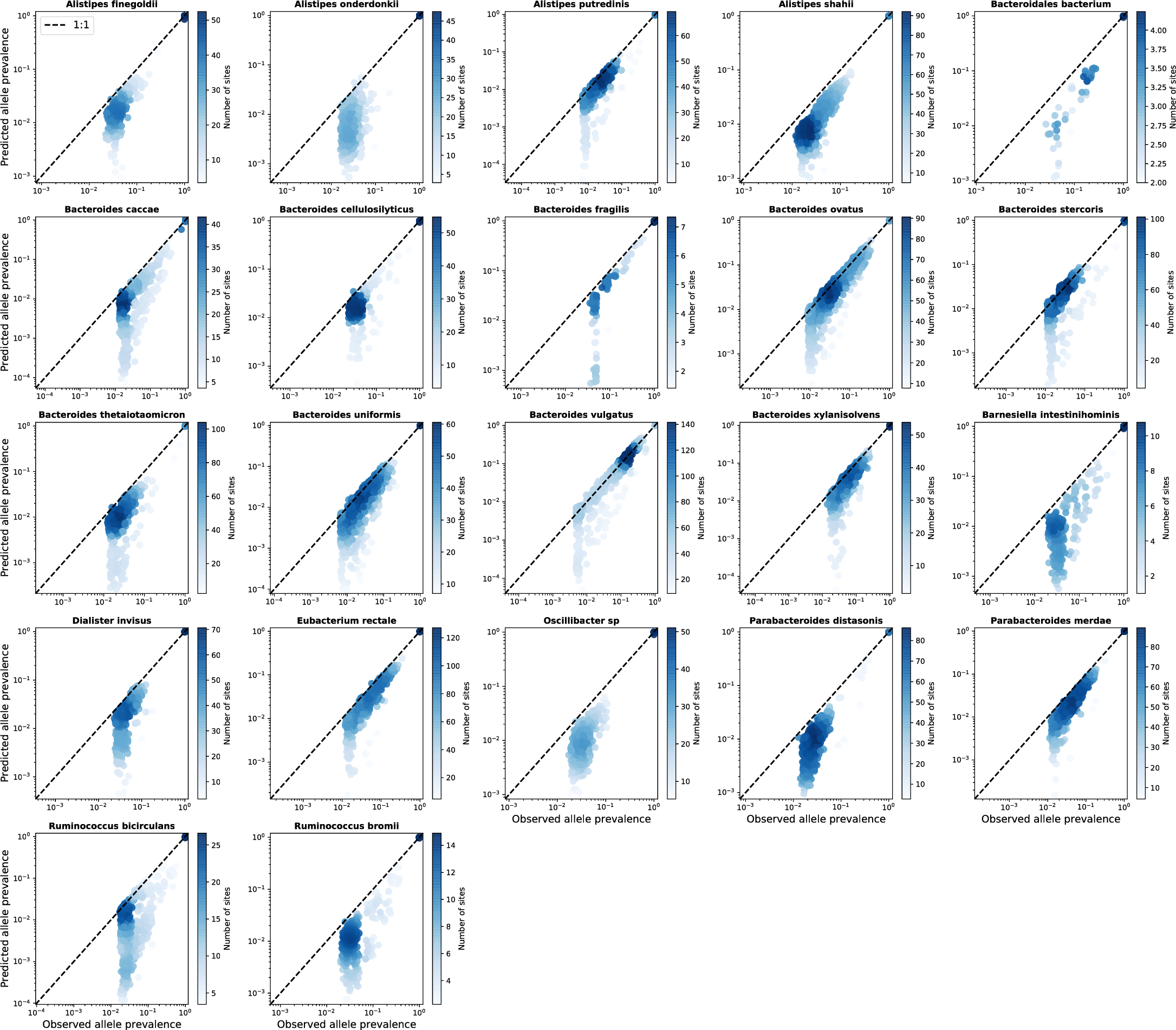

Supplement: S5 Fig — A direct comparison between the observed prevalence of all alleles and their corresponding predicted prevalences using the SLM for synonymous sites. A total of 1,000 datapoints were sampled without replacement for each subplot. (TIF) [file pone.0288926.s005.tif]

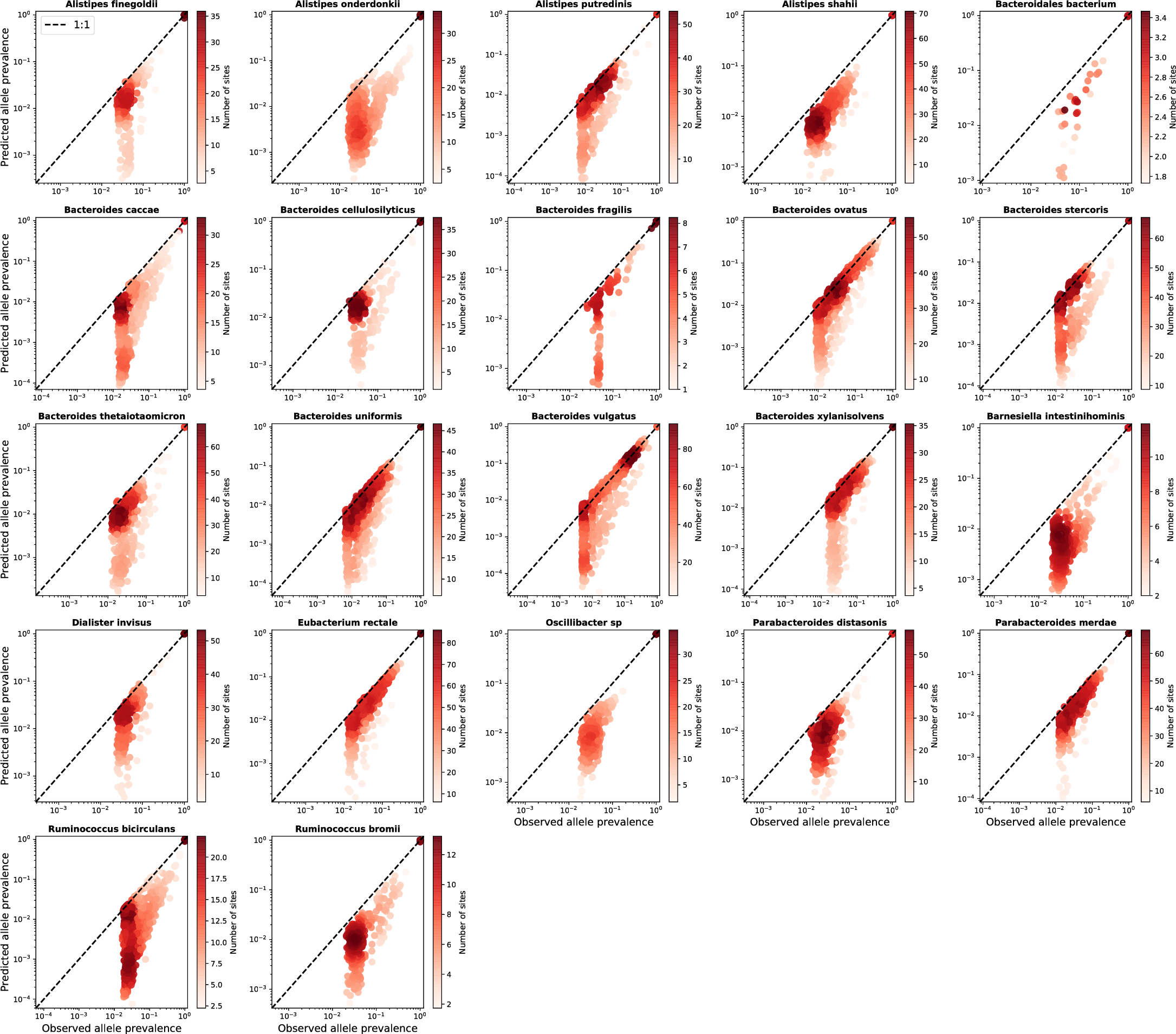

Supplement: S6 Fig — Analogous analyses to S5 Fig using nonsynonymous sites. (TIF) [file pone.0288926.s006.tif]

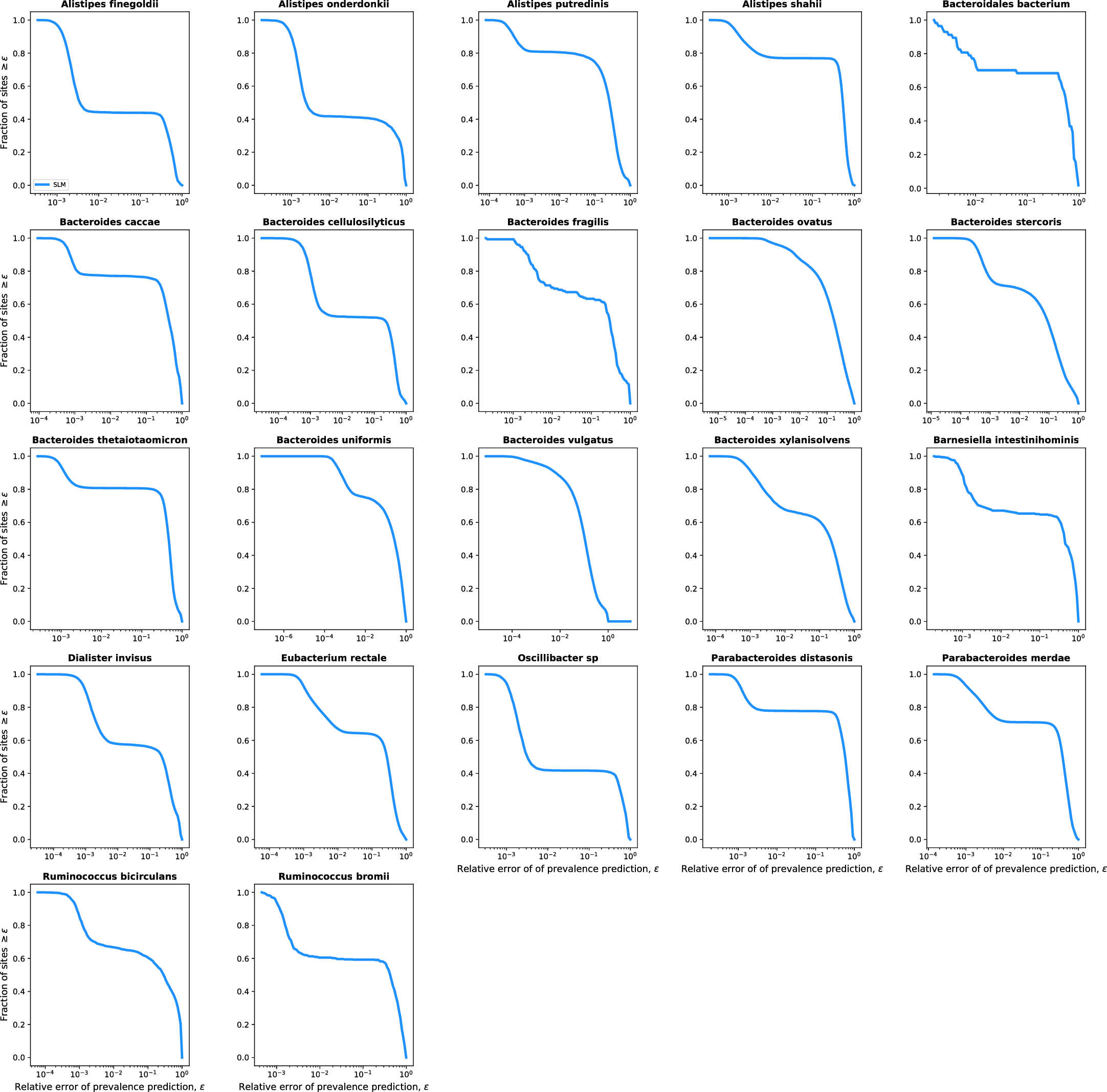

Supplement: S7 Fig — By calculating the relative error of all alleles for the SLM I can examine the error distributions across species. To visually compare the two models, I examined the survival distribution of the relative errors (i.e., the compliment of the empirical cumulative density function). All alleles in this plot are at synonymous sites. (TIF) [file pone.0288926.s007.tif]

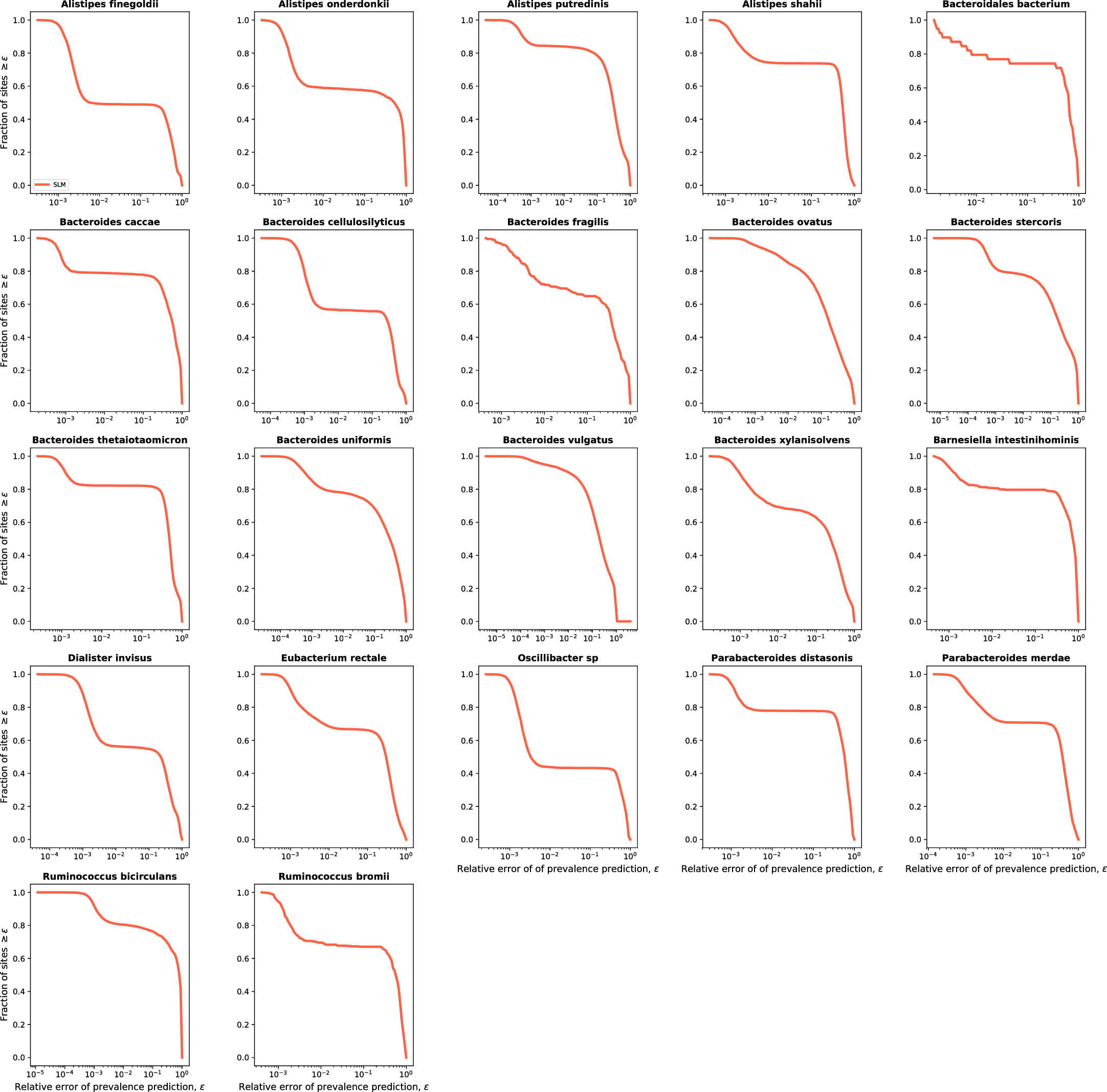

Supplement: S8 Fig — Analogous analyses to S7 Fig using nonsynonymous sites. (TIF) [file pone.0288926.s008.tif]

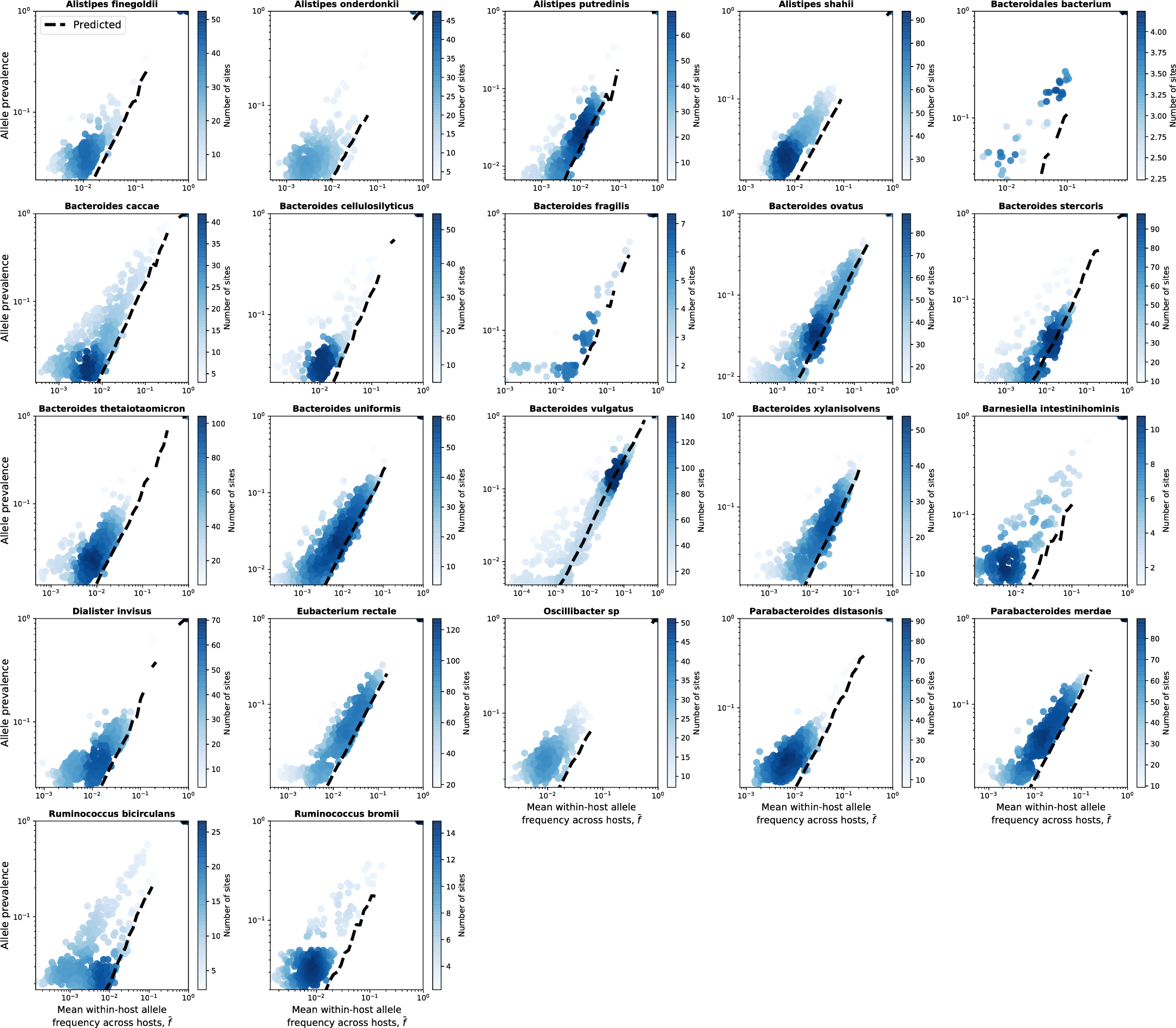

Supplement: S9 Fig — The empirical relationship between the mean frequency of an allele (f¯) and its prevalence across hosts can be recapitulated by the SLM for synonymous sites. Blue dots represent observed values and the shade of blue is proportional to the density of observations. The black line is the predicted relationship calculated using Eq 11. A total of 1,000 datapoints were sampled without replacement for each subplot. (TIF) [file pone.0288926.s009.tif]

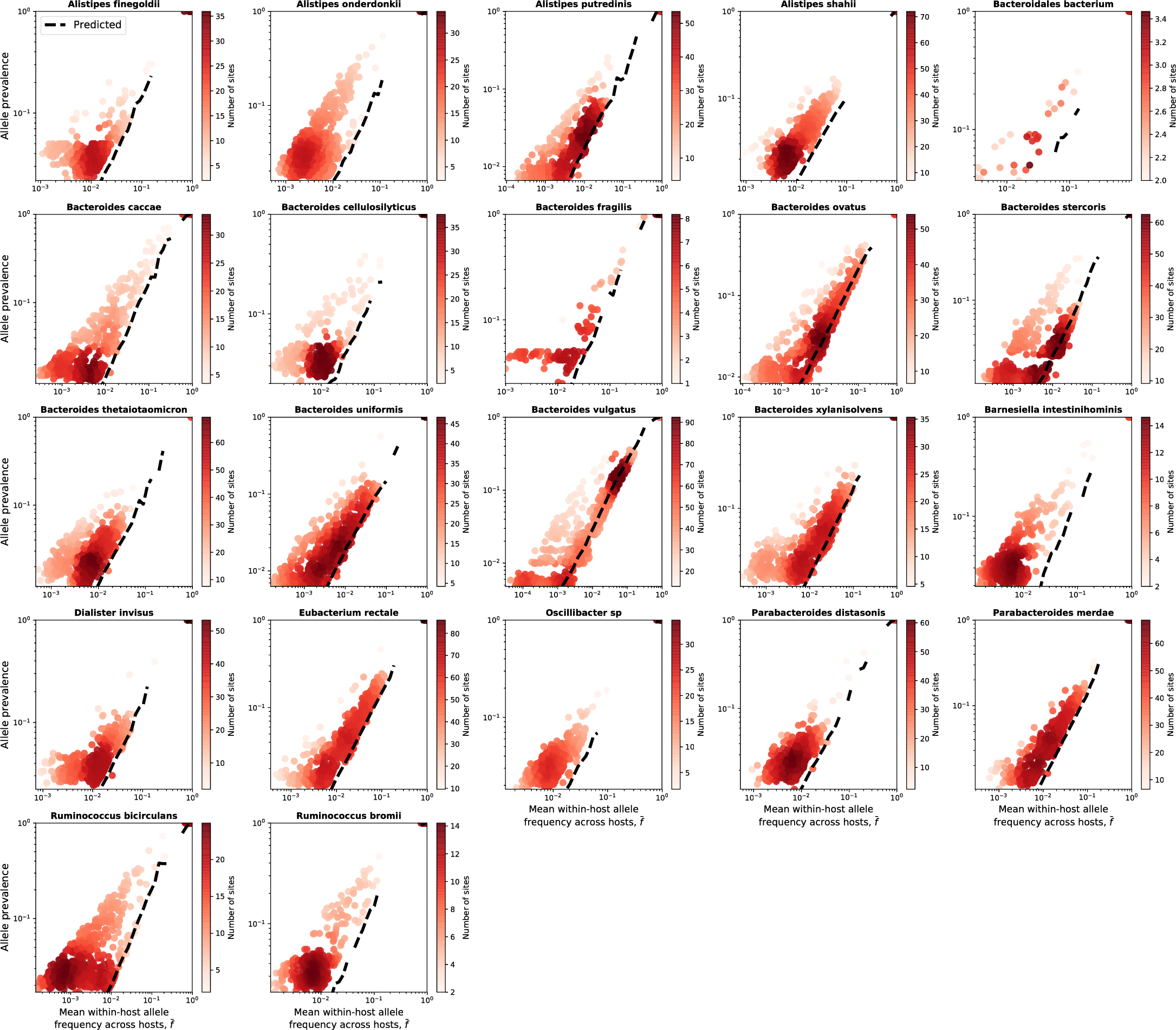

Supplement: S10 Fig — Analogous analyses to S9 Fig using nonsynonymous sites. (TIF) [file pone.0288926.s010.tif]

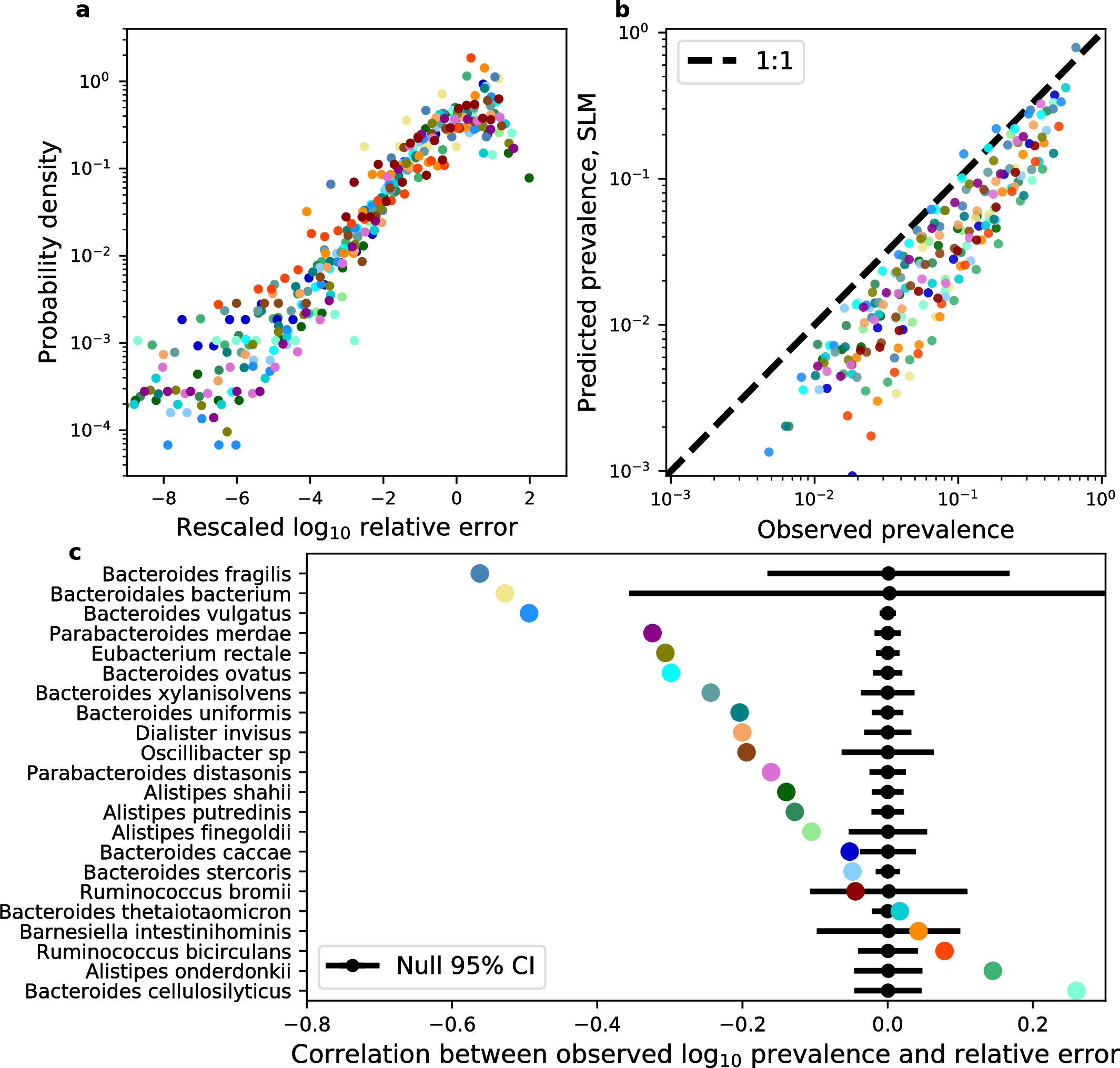

Supplement: S11 Fig — The equivalent analyses in Fig 3 were performed on alleles at nonsynonymous sites. The results of these analyses are qualitatively consistent with those of synonymous sites. (TIF) [file pone.0288926.s011.tif]

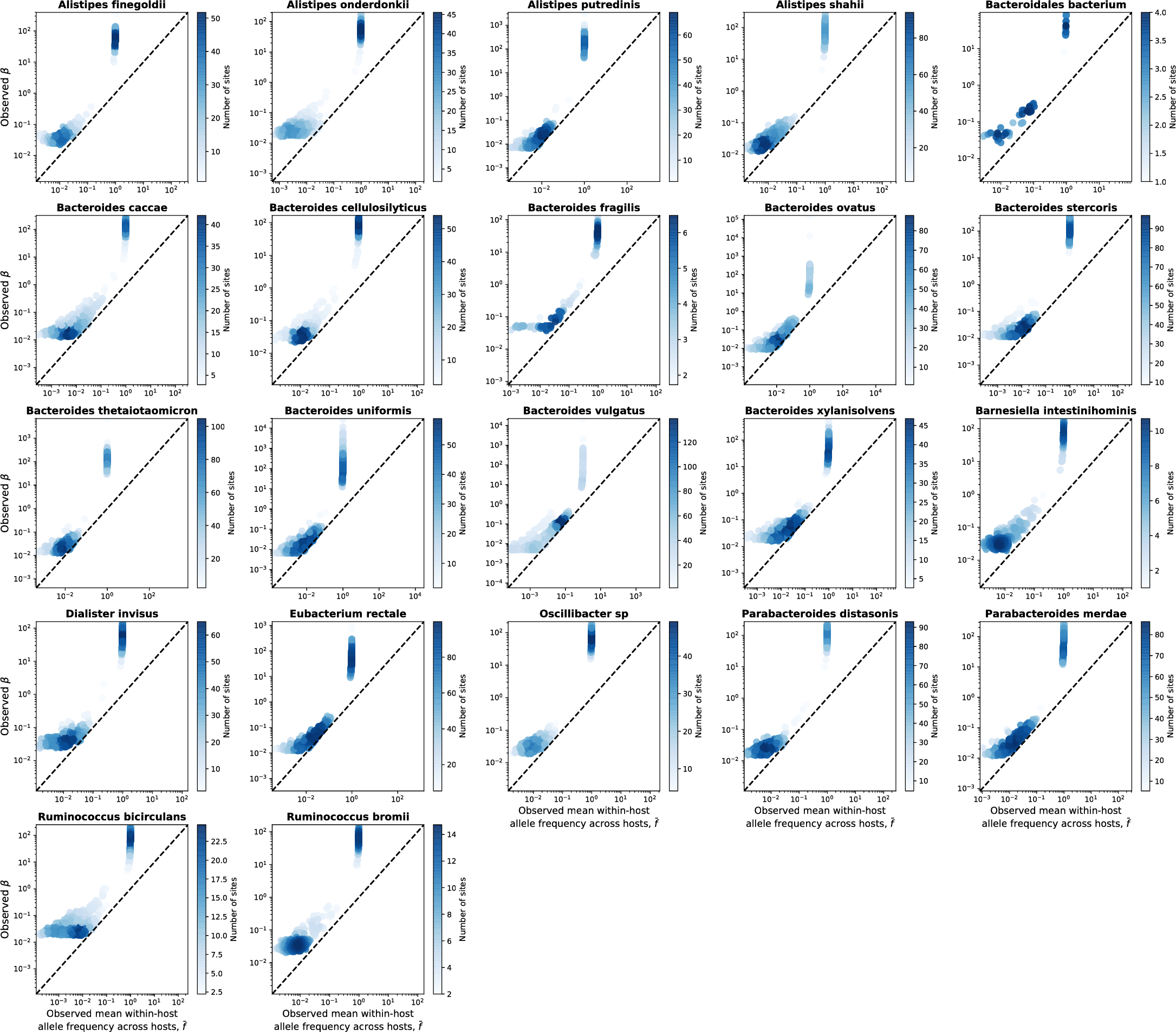

Supplement: S12 Fig — The relationship between the empirical estimates of the two parameters of the SLM: the mean allele frequency across hosts (f¯) and the squared inverse of the coefficient of variation of frequencies across hosts (β). Each point is an individual allele. All alleles are on synonymous sites. A total of 1,000 datapoints were sampled without replacement for each subplot. (TIF) [file pone.0288926.s012.tif]

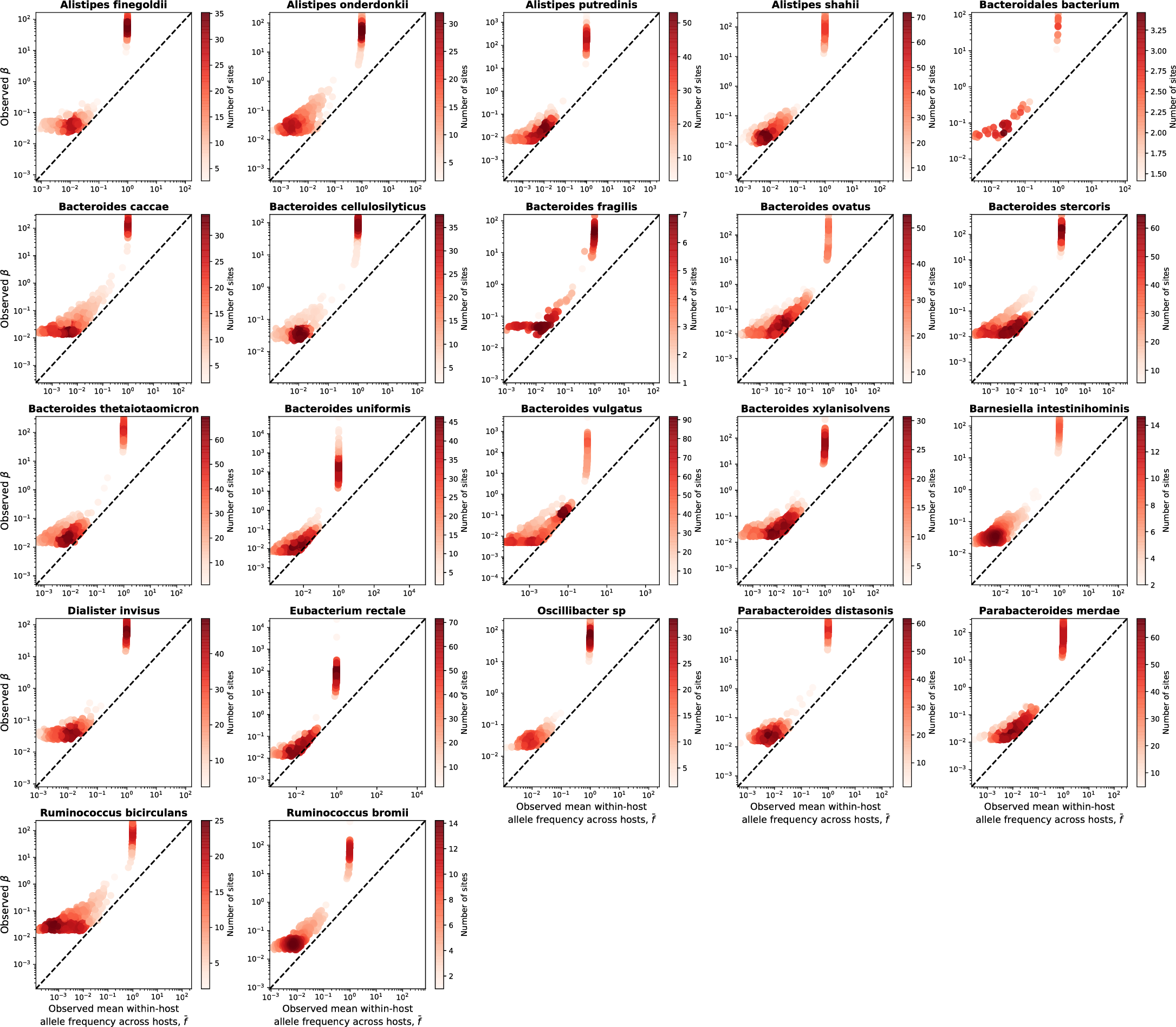

Supplement: S13 Fig — Analogous analyses to S12 Fig using nonsynonymous sites. (TIF) [file pone.0288926.s013.tif]
