# Supplementary material for: A macroecological perspective on genetic diversity in the human gut microbiome
Source: PLoS One. 2023 Jul 21;18(7):e0288926. doi: 10.1371/journal.pone.0288926 (PMC10361512; doi:10.1371/journal.pone.0288926)
Supplement: S1 Text — Derivation of the distribution of allele frequencies under a linearized single-locus model of evolution. (PDF) [file pone.0288926.s014.pdf]

S1 Text

The distribution of allele frequencies under a linearized single-locus model of evolution

Deriving the distribution of allele frequencies in the linear purifying selection regime

The derivation of the gamma distribution for a single-locus model of purifying selection in the low frequency limit has previously been described [1]. We begin with a Langevin equation describing evolution on a single-locus with forward and backward mutation (μ, υ) and selection (s) in a population of N individuals

$$\frac{\partial f}{\partial t} = -sf(1-f) + \mu(1-f) + \upsilon f + \sqrt{\frac{f(1-f)}{N}}\eta(t) \tag{S1}$$

where $\eta(t)$ is a Gaussian noise term. Assuming that $f \ll 1$, the nonlinear selection and back mutation terms drop out and our equation reduces to

$$\frac{\partial f}{\partial t} = -sf + \mu + \sqrt{\frac{f}{N}}\eta(t) \tag{S2}$$

Where I again used the Itô $\leftrightarrow$ Fokker-Planck equivalence [2] to formulate a PDE for the probability $p(f,t)$ that an allele has frequency f at time t

$$\frac{\partial p(f,t)}{\partial t} = -\frac{\partial}{\partial f}\left[\left(-sf+\mu\right)p(f,t)\right] + \frac{1}{2N}\frac{\partial^2}{\partial f^2}\left(fp(f,t)\right) \tag{S3}$$

Solving for the stationary distribution, one obtains a gamma distribution of allele frequencies

$$p(f) = \frac{1}{\Gamma\left(2N\mu\right)}\left(2N|s|\right)^{2N\mu}\exp\left[-2N|s|f\right]f^{2N\mu-1} \tag{S4}$$

Deriving the distribution of allele frequencies in the linear positive selection regime

We again take the low-frequency limit of the single-locus Langevin, but with $s > 0$. This derivation has previously been derived [3,4], which I rederive below for the convenience of the reader.

$$\frac{\partial f}{\partial t} = sf + \mu + \sqrt{\frac{f}{N}}\eta(t) \tag{S5}$$

This SDE can be solved using the method of characteristics. The solution to this problem has previously been derived [3,4], which I rederive below for the sake of completeness. To gain a more comprehensive understanding, the unfamiliar reader should refer to the source material. To work with Eq. S5, it will be convenient to define the moment generating function

$$H(z,t) \equiv \left\langle e^{-zf(t)}\right\rangle \tag{S6}$$

which obeys the following PDE

$$\frac{\partial H}{\partial t} = \left[sz - \frac{z^2}{2N} \right] \frac{\partial H}{\partial z} - \mu z H \tag{S7}$$

We then define the log of the moment generating function along the characteristic curve in the absence of mutation ($\mu = 0$) z^* as

$$\Psi(t) \equiv \log \left[H(z^*(t_f - t), t_f - t) \right] \tag{S8}$$

where t_f is the current time and $H(z^*(t_f - t), t_f - t)$ is a reverse time function. The function $\Psi(t)$ satisfies the following ordinary differential equation and respective initial conditions, where $\varphi(t) \equiv z^*(t_f - t)$

$$\frac{\mathrm{d}\Psi(t)}{\mathrm{d}t} = \mu \varphi(t) \tag{S9a}$$

$$\Psi(0) = \log \left[H(\varphi(0), t_f) \right] \tag{S9b}$$

$$\Psi(t) = \log \left[H(\varphi(t), 0) \right] = 0 \tag{S9c}$$

which gives us the following function and corresponding moment generating function

$$\Psi(t) = \Psi(0) + \int_0^t \mu \varphi(t^{'}) \mathrm{d}t^{'} \tag{S10a}$$

$$H(z, t) = \exp \left[-\mu \int_0^t \varphi(t^{'}) \mathrm{d}t^{'} \right] \tag{S10b}$$

At this point, it is necessary to solve $\varphi(t)$, which requires us to look at Eq. S7 for the $\mu = 0$ case. To derive a solution for this case one must identify the family of curves, z^*, where $\frac{\mathrm{d}H(z^*,t)}{\mathrm{d}t} = 0$. The line $z^* = 2Ns$ is one characteristic curve. Using the chain rule, one can derive an expression for the ordinary differential equation of H

$$\frac{\mathrm{d}H(z^*, t)}{\mathrm{d}t} = \frac{\partial H}{\partial z^*} \frac{\mathrm{d}z^*}{\mathrm{d}t} + \frac{\partial H}{\partial t} = \frac{\partial H}{\partial z^*} \left[\frac{\mathrm{d}z^*}{\mathrm{d}t} + sz^* - \frac{(z^*)^2}{2N} \right] \tag{S11}$$

Solving then the function is equal to zero, one obtains an ordinary differential equation for z^*

$$\frac{\mathrm{d}z^*}{\mathrm{d}t} = -sz^* + \frac{(z^*)^2}{2N} \tag{S12}$$

We can then use our definition of φ and solve

$$\frac{\mathrm{d}\varphi(t)}{\mathrm{d}t} = s\varphi(t) - \frac{\varphi(t)^2}{2N} \tag{S13a}$$

$$\varphi(t) = \frac{z e^{st}}{1 + \frac{z}{2Ns}\left(e^{st} - 1\right)} \tag{S13b}$$

Finally, one can substitute in the solution for $\varphi(t)$ into Eq.S10a and solve the integral

$$H(z,t) = \exp\left[-\mu \int_0^t \frac{z e^{st'}}{1+\frac{z}{2Ns}\left(e^{st'}-1\right)} \mathrm{d}t'\right] \quad \text{(S14a)}$$

$$= \exp\left[-2N\mu \log\left[1+\frac{z}{2Ns}\left(e^{st}-1\right)\right]\right]\Bigg|_0^t \quad \text{(S14b)}$$

$$= \exp\left[-2N\mu \log\left[1+\frac{z}{2Ns}\left(e^{st}-1\right)\right]\right] \quad \text{(S14c)}$$

$$= \left(1+z\cdot\frac{e^{st}-1}{2Ns}\right)^{-2N\mu} \quad \text{(S14d)}$$

Which is the moment generating function for the gamma distribution, providing us with a probability distribution of allele frequencies

$$p(f)df = \frac{df}{f_{max}^{2N\mu}\,\Gamma(2N\mu)} f^{2N\mu-1} e^{-f/f_{max}} \quad \text{(S15)}$$

where $f_{max} \equiv \frac{e^{st}-1}{2Ns}$ [5]. In the neutral limit $s \to 0$, f_{max} reduces to $t/2N$. This result is corroborated by previous work, where that the maximum size that a neutral mutation can reach over $\sim t$ generations is $\sim t/N$ [5]. This result means that the presence or absence of positive selection alone does not invalidate the gamma distribution as a model for $p(f)$ in the $f \ll 1$ limit.

References

1. Nei M. The frequency distribution of lethal chromosomes in finite populations. Proceedings of the National Academy of Sciences of the United States of America. 1968;60(2):517–524.

2. Gardiner CW. Stochastic methods: a handbook for the natural and social sciences. 4th ed. No. 13 in Springer series in synergetics. Berlin Heidelberg: Springer; 2009.

3. Good BH. Linkage disequilibrium between rare mutations. Genetics. 2022;220(4):iyac004. doi:10.1093/genetics/iyac004.

4. Good BH. APHYS 237/BIO251: Quantitative evolutionary dynamics and genomics; 2021.

5. Fisher DS. Course 11 Evolutionary dynamics. In: Les Houches. vol. 85. Elsevier; 2007. p. 395–446. Available from: `https://linkinghub.elsevier.com/retrieve/pii/S0924809907800187`.
