# Supplementary material for: A macroecological perspective on genetic diversity in the human gut microbiome
Source: PLoS One. 2023 Jul 21;18(7):e0288926. doi: 10.1371/journal.pone.0288926 (PMC10361512; doi:10.1371/journal.pone.0288926)
Supplement: Attachment — Submitted filename: 20230420_1538_Response.pdf [file pone.0288926.s015.pdf]

Editor Comments

Thank you for submitting your manuscript to PLOS ONE. After careful consideration, we feel that it has merit but does not fully meet PLOS ONE's publication criteria as it currently stands. Therefore, we invite you to submit a revised version of the manuscript that addresses the points raised during the review process.

In particular, there are a number of writing issues to be addressed, and how the manuscript presents new results that contribute to the field.

> **Author response: We thank the editor for the feedback along with the opportunity to revise and improve the manuscript. In my response I address how all issues raised by both reviewers were addressed in my resubmission.**
>
> **Because my original submission was done using a PDF compiled with latex, I have marked in the original PDF where edits were made for the revision. I have used the LaTeX template provided by PLOS ONE to write the revision: https://journals.plos.org/plosone/s/latex.**
>
> **I have included this detail in my resubmission, but I would like my Funding Statement to read as follows: "This work was supported by the NSF Postdoctoral Research Fellowships in Biology Program under Grant No. 2010885 (W.R.S.)."**
>
> **Regarding the "Reviewer's Responses to Questions" section, I would like to point out that Reviewer 1 marked "No" when asked whether I have made all data underlying my findings fully available. This study solely used publicly available raw data (i.e., FASTQ files) from the Human Microbiome Project: https://aws.amazon.com/datasets/human-microbiome-project/. In keeping with my open science values, I made all processed data that was used to make the figures and perform my analyses publicly available on Zenodo *before* I submitted this manuscript to PLOS ONE: https://doi.org/10.5281/zenodo.6793770. All code that was written to run this project was also made publicly available along with a corresponding Python conda environment before I submitted the manuscript. Since receiving the review, I have worked to increase transparency by expanding the README file of my GitHub repo by adding explicit instructions for rerunning the analyses and revising my code to help with reproducibility. I have also transferred ownership of the repository to my scientific account: StrainPrevalence. Nevertheless, all data and code were made public *before* I submitted the manuscript so I do not know what Reviewer 1 could be referring to as they did not cite any data availability issues in their comments.**

Reviewer #1
This manuscript by William Shoemaker explores intraspecies diversity within and across individual microbiomes, with a focus on the applicability of stochastic logistic model and the existence of strain structure. It is my understanding that the main result of this paper is that the result of this modeling is that strains (ecology) are responsible for more within-host variants than mutation (evolution). This result echoes the current thinking in the field, which has been shown before by other papers, including at least one on which this author is a contributor.

> **Author response: The main result of this paper is that a model of ecology that succeeded at capturing the temporal dynamics of strains *within* a single host (Wolff et al., 2023), the Stochastic Logistic Model of growth, was capable of quantitatively predicting patterns of allelic prevalence *across* hosts for a considerable fraction of sites. In the revision I have rewritten portions of the Abstract, Introduction, and Discussion to emphasize these points (Lines 69-72; 73-83; 441-456).**

This manuscript is difficult to read – partly because what is done is frequently described before what is the goal of this work is, and partly because concepts are a bit muddled / poorly introduced. I list some examples below. I also have some issues with data analysis. Most critically, it is hard to understand what is new in this paper. While it is my understanding that novelty is not a criterion for publication, more fully placing work in the context of the literature is very important for any theoretical or modeling based paper.

> **Author response: Interdisciplinary research is often difficult to communicate. In writing the submitted version of this manuscript I meticulously incorporated feedback from peers in macroecology, microbial ecology, statistical physics, and population genetics. I understand that attempting to please too many points of view can lead to a message that is less than precise. I have worked to revise this manuscript to address the Reviewer's comments. For example, the Abstract now clarifies the purpose of this study and how it fits into the field's current understanding of strain ecology. The Introduction now explicitly states how we can quantitatively describe the dynamics of strains *within a single human host*, a result that provides the motivation to use the SLM to investigate patterns of genetic diversity *across hosts* (Lines 50-83).**
>
> **By adopting a statistical physics approach and leveraging my past efforts to characterize patterns of diversity within a single host, I was able to 1) successfully predict the prevalence of alleles across hosts for a high proportion of sites and 2) provide evidence that the success of my predictions was due to the existence of strain structure.**

Writing issues:
It hard to understand from the abstract, introduction, text, and discussion, exactly is new in this manuscript. The introduction spends much time opining on the value and need for models, without really explaining what has been shown before about the SLM in microbiomes. In

particular, the manuscript cites works, including from this author, showing that strains are responsible for most intraspecies dynamics in the microbiome (this has been known for quite some time) and SLMs describe these dynamics well. What was the open question the other set out to solve? If there was some doubt about the applicability of the SLM model and strains prior to this work, what was the critical missing analysis? What required the need for this approach?

> **Author response: The question was whether an ecological model that succeeded in predicting macroecological patterns at the species level and in predicting the temporal dynamics of strains within a single host could also predict patterns of genetic diversity *across hosts* due to the existence of strain structure.**
> **Making the jump from within-host dynamics to across-hosts patterns is not an easy task at the strain level. Within a given host, the relative abundance of a strain often has to be *inferred* using the temporal trajectories of individual genetic variants (Roodgar et al., 2021; Wolff et al., 2023). This approach requires several metagenomic sequencing samples within a host over a period time, a fairly high cost that limits the number of hosts that can be sequenced. Therefore, when investigating questions of strain macroecology *across a large number of unrelated hosts* you often only have access to samples obtained from a single timepoint (e.g., the Human Microbiome Project data examined in this study). In this scenario, it is reasonable to examine individual nucleotide sites and determine whether their patterns follow what you would expect if they were present because they were on a strain, rather than dealing with the difficulty of inferring strain relative abundances from a single metagenomic sample.**
>
> **I have revised the Introduction to clarify existing knowledge gaps of strain ecology in the human gut. I now explicitly list patterns that the SLM has previously captured at the species level within and across human hosts (e.g., results described in Grilli, 2020) and how it has succeeded at explaining the temporal dynamics of strains *within* a single human host (Wolff et al., 2023). This body of work raises the question of whether the SLM can capture patterns of strain level diversity *across* hosts. Because genetic variants are the constituents of strains, this question can be tested as a question of allelic patterns across hosts and whether the SLM succeeds (Lines 69-72).**

It requires a lot of work for a reader to understand what is being referred to at each point in the text. For example:

1) At Line 94: “the empirical distribution” is unclear; “the distribution of within-host allele frequencies across all hosts” would be clearer. Even clearer would be “the distribution of within-host frequencies at a given site, across all hosts” or something else.

> **Author response: When referring to a distribution in the revised manuscript I now specify the quantity of a distribution (e.g., Line 106).**

2) Line 339: "unlikely for the allele frequency fluctuations I was able to observe". Does this refer to fluctuations across hosts, or time? At the very least, a figure panel should be referred to here.

Author response: I thank the Reviewer for pointing out this typo. "Allele frequency fluctuations" should be "range of allele frequencies", as the proceeding sentence discusses the lower bound on allele frequencies that could be inferred after accounting for sequencing error. This typo has been corrected in the revised manuscript. The data referred to in this sentence is summarized in the proceeding sentence (Lines 375-378).

3) Line 418: what is a "true strain absence". Is the author suggesting that single strain colonization is rare for all species and strains? This would conflict with the literature (e.g. B fragilis almost always colonizes humans as a single strain, PMID: 30673701), but if it is supported by the data this should be made more clear.

Author response: By "true strain absence" I mean strain structure that we do not observe in a host because it is not in the host, as opposed to strain structure that is present at an abundance in a host that is too low to be detected. The gamma distribution implies that the perceived absence of strain structure is typically driven by insufficient depth of sampling (Grilli, 2020), a conclusion that is consistent with the paper cited by the Reviewer (Garud et al., 2019). I have revised this section of the manuscript to clarify these points (Lines 457-471).

4) Line 104: which variable? Mean and variance across what?

Author response: I rescaled allele frequencies by pooling all sites across all hosts and calculating the mean and variance. I rescaled mean allele frequencies by pooling all sites and calculating the mean and variance. I now clarify these points in the revised manuscript (Lines 114-120; 137-138).

5) Line 500: I'm not sure exactly what point is trying to be made here. If there is already an interpretation of the data (antibiotics wiped out a strain), how does the SLM help?

Author response: The utility of an interpretation depends on the goal of the study. A course of antibiotics is a particularly strong perturbation that would alter the dynamics of strains. Other, arguably less drastic perturbations could be caused by a number of factors (e.g., a host traveling to a different climate, a temporary change in diet, etc.). In these scenarios it would be useful to have an ecological framework to determine whether the dynamics of strains differ from a quantitative null. The gamma distribution obtained from the SLM provides such a null, allowing researchers to determine whether the strain dynamics we observe *truly depart* from what we would expect if growth rate, environmental noise, and carrying capacity

were the only ecologically important factors. For example, the gamma distribution was recently used as a null model to determine whether interactions between strains were sufficiently strong that the assumptions of the SLM were invalid (Goyal et al., 2022). In the revision, I have revised this section of the manuscript to incorporate the Reviewer's feedback and increase overall clarity (Lines 554-558).

Technical notes

6) I find Figure 1 (as well as some s to not be presented in a manner such that I can evaluate the claim being made: that the rescaled distributions are similar across species. To evaluate this, I would like to see example distributions (perhaps cumulative curves) drawn for each species – instead what I am presented with is a mess of dots on top of each other in 1b and 1d. I see notable differences, even though these are rescaled already. No goodness of test fits are applied and no alternative distributions are provided.

Author response: The purpose of Fig. 1b is to provide empirical motivation for examining the SLM as a model of strain ecology *across* hosts. A gamma appears to capture the distribution, so one should investigate models of ecology that generate a gamma distribution. I propose the SLM as such a model. In the revision I have used a standard fitting procedure that I now describe in the manuscript (Lines 120-134). Because the distribution in Fig. 1b uses tens of thousands of data points, a goodness of fit test would inevitably produce a *P*-value less than 0.05, suggesting that a more insightful approach would be to compare the fit of the gamma with alternative distributions.

There are many options for alternative distributions, though the Reviewer did not recommend a specific one. Since the empirical Abundance Fluctuation Distribution (AFD) in Fig. 1c is wide (stretches over several orders of magnitude), a Gaussian would be inappropriate on the outset. As a reasonable alternative, I chose the lognormal as it has been used in the past as a macroecological comparison for the gamma AFD (Grilli, 2020). By fitting a lognormal, we see that it in no way captures the empirical distribution (Fig. 1c). This result is even more apparent when plotting the same data as a survival distribution, as recommended by the reviewer. Here we see that all species except one follow the gamma distribution (Fig. S2 in the revised manuscript, embedded below). I cannot compare these distributions using a likelihood ratio test since they are not nested, but by calculating the Akaike Information Criterion it is clear that the gamma is a far better descriptor of the empirical distribution ($AIC_{gamma} = 6,277,330, AIC_{lognorm} = 6,618,492$; Lines 132-134). The AIC is so high because we are summing a large number of data points for each test.

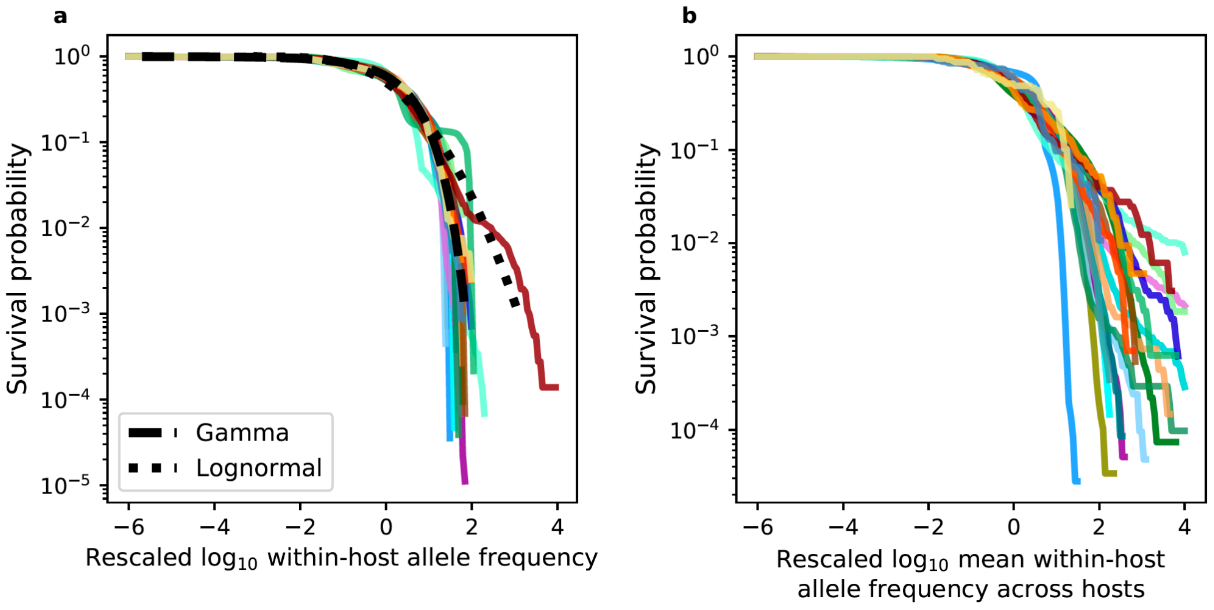

Regarding distributions of mean abundances, I purposefully chose *not* to propose a mathematical distribution (e.g., Gaussian, gamma, etc.) to explain the empirical distribution in Fig. 1d because a mathematical descriptor of the distribution is not relevant to the subsequent analyses. Using the sampling form of the gamma we can only describe the patterns of genetic diversity across hosts for a given site *using the empirical mean and variance*. We are not interested in suggesting a probability distribution to capture the empirical distribution of means because the mean is an empirical input used to calculate the predicted value of prevalence (Eq. 6). This point has been clarified in the revised manuscript (Lines 143-152).

Regarding the appropriateness of the gamma, I explicitly test the gamma distribution by using the mean and variance to predict the prevalence of an allele across hosts (Fig. 2-4). This is a prediction obtained using only empirical inputs (i.e., the mean and variance) with *zero free parameters.* Alternatively stated, *no statistical fits were performed for that analysis*.

7) Site frequency spectra are not widely used in microbiology, as the SFS is something that results from recombination. The idea that within-host or across-host evolution would result in a meaningful SFS is confusing to this reviewer.

Author response: The SFS has been intensively examined in theoretical studies motivated by microbial evolution, including studies that examine purely asexually evolving populations (Cvijović et al., 2018; Kosheleva & Desai, 2013; Neher & Hallatschek, 2013; Okada & Hallatschek, 2021) as well as populations evolving with varying rates of recombination (Good et al., 2014; Neher et al., 2013). Furthermore, the SFS has been the primary data object used for strain inference, whether it is by

quantifying the existence of strain structure (e.g., StrainFinder in Smillie et al. (2018)) or by obtaining a single haplotype from the SFS using the unique form generated by the existence of co-occurring strains (Garud, Good et al., 2019).

The reviewer is correct in that one's interpretation of the SFS depends on the degree of recombination in the population. There is increasing evidence that bacteria recombine more often than previously thought. The decay of measures of correlation in genotype frequencies (e.g., linkage disequilibrium) decays with genetic distance (i.e., # base pairs) at a faster rate than expected by an asexual population (Garud, Good et al., 2019; Good, 2022). While this rate of decay is slower than what might be expected under free recombination, it does mean that an asexual view of bacterial evolution, and how that view shapes our understanding of empirical patterns such as the SFS, is not entirely valid.

However, in all these studies, and in the field at large, the SFS is defined as the distribution of allele frequencies among all sites *within* a single host/population. In this study I solely refer to the distribution of allele frequencies at a single site *across* hosts, a distribution that has previously been defined as the single-site frequency spectrum (Theys et al., 2018).

8) Fig 2c: I'm not sure what the value of predicting "mean within-host allele frequency across hosts" is, if all hosts—including those without the allele at all—are included. Perhaps I am missing something. Also, it seems like most of the data here isn't fitting the line, but instead fitting a curve starting at y = 10^-1. These graphs should be made as density maps to make it clearer how well the data and the prediction line up.

Author response: In this manuscript I predict the fractions of hosts harboring a given allele (i.e., prevalence). I do not predict the mean frequency of an allele across hosts, that quantity is an empirical input used to calculate the predicted prevalence (Eq. 6). Regarding why we want to include hosts where an allele was not observed in our calculation of the mean, we are hypothesizing that allele frequencies across hosts can be described using a gamma distribution, the stationary solution of the SLM. If the underlying empirical data truly followed the form of the gamma distribution I used that explicitly accounts for total depth of coverage (i.e., sampling; Eq. 4), then excluding hosts where an allele was not observed (i.e., the allele had a coverage of zero) means that I would no longer be sampling from that distribution, I would instead be sampling from a *truncated* form of that distribution.

Regarding the data, first, the black dashed line is a prediction using measures obtained from empirical data (i.e., the mean and variance) with zero free parameters. It is not a statistical fit because there were no free parameters to infer. I now clarify this point throughout the manuscript, including at lines that cite Fig. 2c (Lines 77-80; 277-279; 657-660), and the figure legend.

Second, I stated in the original submission that the prediction tended to succeed for alleles with high mean frequency. We expect that some fraction of polymorphic sites for a given species within a host are polymorphic because they are on the background of a strain (i.e., ecology), whereas other sites are segregating due to evolution. This result is consistent with the conceptual picture that both ecology and evolution are operating within a given species, but it is novel in that I was able to predict the prevalence of alleles with a high mean abundance across hosts using an ecological model that succeeded at predicting the dynamics of strains within hosts (Wolff et al., 2023). I now clarify these points in the revised manuscript (Lines 441-456).

The figures presented in the original manuscript were already illustrated as density maps. The color of each data point is proportional to the density of points in its immediate area. However, this detail was not made explicit, so the revised manuscript now explicitly mentions this detail in the figure legend. There was a plotting error in the original manuscript, as the color did not correspond to the density. This has been corrected in the revised manuscript. I have also added a color bar indicating how the color corresponds to the number of points for all figures with density plots (Figs. 2, S5, S6, S9, S10, S12, S13).

Some thoughts: It is clear that the author has put a lot of work into the generation of this data and model, but it is always important to be clear about what is known and what is novel. The formalization of an already proposed model can be valuable. Presenting a model which more clearly shows what has been shown by others could also be valuable; if this is the goal than more effort needs to be put into making it clear.

Author response: I agree with the reviewer. The novelty of this study is that I demonstrated how an ecological model that captures the dynamics of strains within a single host (Wolff et al., 2023) was able to capture patterns of genetic diversity across hosts due to the existence of strain structure. In the revision I have incorporated their feedback by carefully clarifying in the Abstract, Introduction, and Discussion the novelty of this work and how it builds on prior studies (Lines 56-72; 73-83; 441-456).

Reviewer #2

Major comments

Generally, this is an interesting work. However, as someone with a limited background in biostatistics, I find the description of the underlying modeling approach very hard to follow. For instance, the assumptions behind the SLM described from line 197-208 are not clear to me. Hence, my recommendation is to clarify the description of the SLM, its assumptions, and the overall discussed concepts for readers less familiar with them. I understand that this is not my

area of expertise, but it would be very helpful to make the presented results more accessible to scientists in the gut microbiome field.

> **Author response: I thank the reviewer for their comments. I have worked to clarify the motivation for and definition of the SLM in the revised manuscript. I have added brackets in Eq. 1 to describe the physical meaning of each term in the equation and additional text to describe how noise is modeled in the equation (Lines 211-242). Additional technical steps are provided in the Materials and Methods (Lines 633-679). As for the rest of the manuscript, I have revised several sections in the Abstract, Introduction, and Discussion to clarify novelty of this work and how it builds on prior studies (Lines 56-72; 73-83; 441-456).**

Minor comments

1) The legends for Figures 1-4 are more a repetition of the Results section than a clear description of what is shown in each panel.

> **Author response: I initially included additional descriptive details in the legends at the request of past reviewers. The legends of Fig. 1-4 have now been revised to emphasize the contents of the figures rather than the results.**

2) Line 339: MAGPD is mentioned here for the first time without having been defined before.

> **Author response: A definition of the acronym has now been included and moved earlier in the manuscript to where data from MAPGD was first described (Line 97).**

3) Line 448-449: “Regardless of the specific model, the conclusion that a substantial fraction of observable alleles are primarily subject to ecological, as opposed to evolutionary, dynamics is of consequence to studies of genetic diversity in the human gut.” It is not clear to me which part of the Results section precisely shows this.

> **Author response: In this manuscript I demonstrate that the Stochastic Logistic Model of growth, an ecological model that has been used to capture the temporal dynamics of strains within a single host, can predict patterns of allelic prevalence *across hosts* for alleles of moderate-to-high mean frequency (Fig. 2, 3). Because alleles are the constituents of strains, this result means that the *across-host* patterns of a substantial portion of alleles are driven by strain-level ecology. This result is supported by the observation that the error of the prevalence predictions is inversely related to the fraction of hosts harboring strains (Fig. 4). I have revised the Discussion to clarify this point (Lines 441-456).**

4) Line 655: There is a figure legend for Figure 5, which does not exist.

Thank you for pointing this out. The figure legend has been removed and there never was an intention of having a fifth figure. It was an artifact of reformatting the paper in multiple latex templates.

5) Line 704: Is this really the correct URL for the Human Microbiome Project?

Author response: I have used a URL that links to a repository containing the data of the Human Microbiome Project. This URL and the data therein were listed under the Data and Code Availability Statement of a previously published paper that also used Human Microbiome Project data (Garud, Good, et al., 2019). In the revision I now include the official URL of the Human Microbiome Project alongside this link.

References

Cvijović, I., Good, B. H., & Desai, M. M. (2018). The Effect of Strong Purifying Selection on Genetic Diversity. *Genetics*, *209*(4), 1235–1278. https://doi.org/10.1534/genetics.118.301058

Garud, N. R., Good, B. H., Hallatschek, O., & Pollard, K. S. (2019). Evolutionary dynamics of bacteria in the gut microbiome within and across hosts. *PLOS Biology*, *17*(1), e3000102. https://doi.org/10.1371/journal.pbio.3000102

Good, B. H. (2022). Linkage disequilibrium between rare mutations. *Genetics*, *220*(4), iyac004. https://doi.org/10.1093/genetics/iyac004

Good, B. H., Walczak, A. M., Neher, R. A., & Desai, M. M. (2014). Genetic Diversity in the Interference Selection Limit. *PLOS Genetics*, *10*(3), e1004222. https://doi.org/10.1371/journal.pgen.1004222

Goyal, A., Bittleston, L. S., Leventhal, G. E., Lu, L., & Cordero, O. X. (2022). Interactions between strains govern the eco-evolutionary dynamics of microbial communities. *ELife*, *11*, e74987. https://doi.org/10.7554/eLife.74987

Grilli, J. (2020). Macroecological laws describe variation and diversity in microbial communities. *Nature Communications*, *11*(1), 4743. https://doi.org/10.1038/s41467-020-18529-y

Kosheleva, K., & Desai, M. M. (2013). The Dynamics of Genetic Draft in Rapidly Adapting Populations. *Genetics*, *195*(3), 1007–1025. https://doi.org/10.1534/genetics.113.156430

Neher, R. A., & Hallatschek, O. (2013). Genealogies of rapidly adapting populations. *Proceedings of the National Academy of Sciences*, *110*(2), 437–442. https://doi.org/10.1073/pnas.1213113110

Neher, R. A., Kessinger, T. A., & Shraiman, B. I. (2013). Coalescence and genetic diversity in sexual populations under selection. *Proceedings of the National Academy of Sciences*, *110*(39), 15836–15841. https://doi.org/10.1073/pnas.1309697110

Okada, T., & Hallatschek, O. (2021). Dynamic sampling bias and overdispersion induced by skewed offspring distributions. *Genetics*, *219*(4), iyab135. https://doi.org/10.1093/genetics/iyab135

Roodgar, M., Good, B. H., Garud, N. R., Martis, S., Avula, M., Zhou, W., Lancaster, S. M., Lee, H., Babveyh, A., Nesamoney, S., Pollard, K. S., & Snyder, M. P. (2021). Longitudinal linked-read sequencing reveals ecological and evolutionary responses of a human gut microbiome during antibiotic treatment. *Genome Research*, *31*(8), 1433–1446. https://doi.org/10.1101/gr.265058.120

Smillie, C. S., Sauk, J., Gevers, D., Friedman, J., Sung, J., Youngster, I., Hohmann, E. L., Staley, C., Khoruts, A., Sadowsky, M. J., Allegretti, J. R., Smith, M. B., Xavier, R. J., & Alm, E. J. (2018). Strain Tracking Reveals the Determinants of Bacterial Engraftment in the

Human Gut Following Fecal Microbiota Transplantation. *Cell Host & Microbe*, *23*(2), 229-240.e5. https://doi.org/10.1016/j.chom.2018.01.003

Theys, K., Feder, A. F., Gelbart, M., Hartl, M., Stern, A., & Pennings, P. S. (2018). Within-patient mutation frequencies reveal fitness costs of CpG dinucleotides and drastic amino acid changes in HIV. *PLOS Genetics*, *14*(6), e1007420. https://doi.org/10.1371/journal.pgen.1007420

Wolff, R., Shoemaker, W., & Garud, N. (2023). Ecological Stability Emerges at the Level of Strains in the Human Gut Microbiome. *MBio*, *0*(0), e02502-22. https://doi.org/10.1128/mbio.02502-22
