# Supplementary material for: A macroecological perspective on genetic diversity in the human gut microbiome
Source: PLoS One. 2023 Jul 21;18(7):e0288926. doi: 10.1371/journal.pone.0288926 (PMC10361512; doi:10.1371/journal.pone.0288926)
Supplement: Attachment — Submitted filename: 20230608_2201_point-by-point.pdf [file pone.0288926.s016.pdf]

Editor Comments

I am in agreement with one of the reviewers, who has given detailed constructive criticism to further improve the manuscript. It is important that the manuscript be carefully revisited to systematically address all the concerns of the reviewers.

> **Author response: I thank the editor for the feedback along with the opportunity to improve the manuscript. In my response I describe how all comments made by both reviewers were addressed in my resubmission.**

Reviewer #1

This manuscript has improved from the last version. In R2R document, the author states that the purpose of this manuscript is to demonstrate that the previously proposed model, in which (1) most intrastrain polymorphisms arise from migration and growth of strains rather than within-person evolution; and (2) this is formalized with an SLM model, can explain allelic prevalence across hosts. Bolstering the SLM model and the importance of strain structure is a fine goal (though I have a question on the contextualization of this below), and now I can begin to interpret the manuscript.

> **Author response: I thank the reviewer for their constructive comments before, which have improved the manuscript.**

Significant work still remains to be done on the writing side. While the author appears to have attempted to address my concerns, shortcuts were taken rather than addressing the real problem (e.g. point #1f below), the text still falls short of communicating what is being advanced by these models, and murky language is used throughout--- such that I still relied heavily on the response to the reviewer (R2R) to interpret what the author intended. I do not think that all interdisciplinary work needs to be so difficult to read. I also have major concerns around the framing and some technical aspects of this work.

1) The writing is murky and it is hard to follow what point the author is making throughout.

> **Author response: In this revision I have gone to considerable efforts to clarify my points. In addition to incorporating the reviewer's suggestions, I have gone through the manuscript and identified additional opportunities for clarification.**

1a) As an example to the author, I provide below an edited alternative of the middle of the abstract which is easier to read. In this example, the connection between strains and ecology is made more clear to a naiive reader, and what the SLM is modeling (strains) is made clear within the sentence that introduces it. The use of a "however" is removed from a location where a clear contrast isn't being made. The phrases "determine whether" and "is capable of capturing" are replaced by more direct language. I am not sure if the suggestion for the last sentence reflects what the author intended; if I am incorrect, the author should clarify what is meant by "patterns

of genetic diversity across hosts follow statistically similar forms".
Revised abstract:
"…Recent efforts have suggested that a large fraction intrahost, intraspecies, genetic variation is driven by dynamics between co-colonizing of strains of the same species, highlighting the importance of modeling ecological forces. In particular, the Stochastic Logistic Model (SLM) of growth, commonly used in macroecology to describe between-species variation, has been used successfully to predict the temporal dynamics of strains within a single human host. Here, using data from common microbial species across a large cohort of unrelated hosts, I show that the SLM also successfully predicts across-host genetic diversity in the human gut. The SLM predicts both the distribution of allele frequencies across hosts and the fraction of hosts harboring an allele for a given site (i.e., prevalence). The accuracy of the SLM in predicting these across-host parameters is correlated with independent estimates of strain structure, confirming that the success of the SLM arises from the presence of strain-level ecology in the human gut.

> **Author response: I appreciate the reviewer's suggestions. I have revised the Abstract to incorporate all suggested edits made by the reviewer.**

1b) In regards to my point #2 in the previous round the authors have changed a phrase but have not fixed the core of the problem, which is requiring a reader to read every sentence in order and guess what is being referred to be able to follow. One should not write "allele frequency fluctuations that I was able to observe" when they could say something like "THE LARGE allele frequency fluctuations ACROSS HOSTS" or whatever feature the author is actually intending here. As a reader, I cannot evaluate the validity of a sentence that is vague.

> **Author response: In the prior round of comments the reviewer quoted a line of the manuscript that contained a typo. I fixed the typo and I thank the reviewer for clarifying their prior comment. The revision now specifies that "fluctuations" refers to "fluctuations across hosts" (line 410). I have also gone through the manuscript and clarified every mention of the term "fluctuation".**

1c) Line 62 would be clearer if the manuscript said "In macroecology, the SLM…. "

> **Author response: I have made the suggested edit (line 69).**

1d) The last paragraph of the introduction would be much clearer with specific methods (is "an ecological model" different than the SLM already discussed? In the last sentence, what is "an alternative evolutionary model"?) or with the general methods removed (just state result).

> **Author response: I have changed "an ecological model" to "the SLM as a model of ecology" (line 84). I have rewritten the last sentence to clarify that I am referring to evolutionary Langevin equations that also generate the same stationary probability distribution as the SLM (lines 91-93). I then specify that I inferred the presence of**

strain structure and found that strain structure was correlated with the accuracy of the SLM in predicting allelic prevalence (lines 93-97).

1e) "First, I examined the distribution of within-host allele frequencies across hosts." This should be explained better. This is not a normal metric, and it would be great if this could be spelled out.

Author response: I have made the requested edits, changing the quoted line to:

"First, I obtained the distribution of across-host allele frequencies for each nucleotide site and then pooled the frequencies of all sites." (line 118-119).

1f) Line 70: "If the typical allele was present due to evolutionary forces, then the empirical distribution of within-host allele frequencies across hosts can be viewed as an ensemble of single-site frequency spectra." By definition I believe that this metric is an ensemble of SFS. I think you mean "… across hosts WOULD BE EQUIVALNT TO THE ensemble of single-site frequency spectra EXPECTED FROM WITHIN PERSON EVOLUTION." It would also be helpful for the reader if the shape of each of these expectations were described in terms of the distributions used in Figure 1 in this paragraph.

Author response: I appreciate the reviewer's comments regarding the importance of clarity. I have incorporated their request into the manuscript (lines 119-123).

The purpose of Fig. 1c is to illustrate how a single probability distribution is capable of explaining the empirical distributions of a many species. Visual inspection (and AIC tests based on the reviewer's previous requests) suggests that the gamma distribution sufficiently captures the empirical distribution. I then identify the SLM as a reasonable model because 1) of its past success in explaining within-host strain dynamics and 2) because its stationary distribution is the gamma distribution. I then proceed with testing the gamma at each site (Figs. 2,3) and determine that the accuracy of the gamma is correlated with an independent estimate of strain structure (Fig. 4), validating the ecological interpretation of the gamma distribution. In the revision I identified sections of the manuscript related to the above points that required additional clarity and made appropriate edits (lines 219-227, 253-255).

1g) The dN/dS curve mentioned is all for Bacteroides and related species. This curve has not been investigated in other major taxa (e.g. Clostridia). In addition, this result is mentioned but not explained well enough for a reader to understand this point. "a clear example being the observation that THE RATIO OF NONSYNONYMOUS DIVERGENCE TO SYNONYMOUS DIVERGENCE DECAYS NEARLY IDENTICALLY OVER LONG TIME SCALES OF across microbial species in the human gut"

Author response: The curve presented in Fig. 3 of Garud, Good et al. was expanded with additional species in Shoemaker et al., where it visualized species from 20

genera, 14 families, 7 orders, 6 classes, and 5 phyla (2019; 2021). The assemblage of species represented in Shoemaker et al. includes members of the class Clostridia (e.g., *Clostridium*, *Butyrivibrio*; 2021). I understand that it is not an exhaustive representation of bacterial diversity, but it provides empirical motivation for identifying patterns that hold across evolutionarily distant species in the human gut microbiome. Therefore, I briefly summarize the taxonomic diversity in the line quoted by the reviewer in the revision. I have also edited the line to state that the ratio *dN/dS* decays with increasing *dS*, where *dS* is interpreted as a proxy for evolutionary time (lines 47-51).

1h) "This disproportionate focus on individual species and differences between species can lead to idiosyncratic notions about the typical dynamics of a genetic variant in the microbiome….. Instead, it is reasonable to start by identifying genetic patterns" I am just not sure what this means. One interpretation of these sentences is that the author is claiming that looking at things one species at a time is incorrect. I'm not sure the author needs to say this. I think it would be enough to say that identifying a single model that works across species would reveal general principles of eco-evo dynamics across microbiomes.

Author response: Fig. 1c-f contains visual examples of what is meant by the line quoted by the reviewer. I believe that it is reasonable to first inspect empirical patterns before applying a model. From past experiences, discussions with peers, and assessing published literature, the practice of plotting distributions of the same quantity from different species/systems on the same axis to identify qualitatively similar behavior is often overlooked in the life sciences. This observation was the motivation for describing the benefits of performing a "data collapse" and investigating whether distributions of within-host allele frequencies for different species have invariant forms (lines 101-107; Fig. 1c). However, the approach that I, and other researchers in microbial evolution and ecology, took is not the singular approach. Rather, it can be viewed as one way to address scientific questions rather than a prescription and I have revised the manuscript to reflect this view and the views of the reviewer (lines 38-45).

There are parts of the results section that are likewise confusing, but I have selected to use my time to give several clear examples of areas for improvement in the introduction and results as an example to the author of how more clarity can be achieved.

Author response: I have combed through the manuscript and done my best to meet their requests. I appreciate the reviewer for identifying areas where clarity can be improved.

Other major concerns:

2) It is very true that new and better eco-evo models are needed for microbial populations, but the presentation of current knowledge in the population genetics field and the remaining gaps are incorrectly summarized.
2a) The surprise of strain structure is a bit overstated. One can infer that strains are a real entity by building phylogenetic trees from available isolates or from metagenomic samples with single strains (e.g., quasiphasable as in Garud and Good et al). This sort of expectation should be more explicitly stated in the introduction.

> **Author response: In this manuscript I start from the observation that a number of genetic variants at the species level neither become fixed nor go extinct over extended periods of time within a single human gut. This empirical motivation has been used in other manuscripts to introduce readers to the concept of multiple strains co-occurring within a single human host (Good & Hallatschek, 2018).**
>
> **In lines 11-16 I altered the language to be more descriptive so that I do not overstate the existence of strain structure. At the request of the reviewer, I have included a sentence discussing how this ecological structure is often reflected in the shape of phylogenetic trees constructed from microbial isolates (lines 13-16).**

2b) Page 2, Line 20: "Such dynamics are a clear departure from those captured by standard population genetic models, where genetic variants either arise in a population due to mutation or are introduced by migration and then proceed towards extinction or fixation (i.e., origin-fixation models), suggesting that measures of genetic diversity estimated within the human gut are shaped by the ecology of strains alongside evolution [10]. " There are many population genetic models that deal with standing variation – the entire field of human population genetics on quantitative traits comes to mind. I think the bigger gap is that models for population genetics on standing variation don't deal with the genome-wide linkage inherent to bacteria.

> **Author response: The existence of strain structure in microbial communities is not a matter of standing genetic variation in the way it is for humans. Microbial strains that are present the same host represent a form of ecological structure. Namely, what we observe as standing genetic variation that persists over extended timescales (over a year in Wolff et al., 2023) is in reality the presence of multiple community members that occupy different ecological roles while *de novo* mutations continue to be acquired and segregate *within each strain* (Dapa et al., 2023; Roodgar et al., 2021).**
>
> **This empirical observation has motivated those in the field to develop models that integrate principles from microbial ecology with those from population genetics. For example, in Good, Martis, et al. the authors develop an evolutionary model where each strain acquires mutations that impact growth rate (i.e., fitness) at one rate and mutations that impact which resources they consume at another rate (i.e., ecological strategy; 2018). This model captures the emergence and coexistence of strains within a human host over time, where each individual strain can be viewed as an asexual population where the population scaled mutation rate (population size multiplied by**

the per-genome mutation rate) is sufficiently small such that typically only a single mutation is segregating within a given strain (strong selection weak mutation limit). Subsequent modeling efforts extend this model to the genome-wide linkage regime where asexually reproducing strains have a population scaled mutation rate sufficiently high so that multiple mutations segregate *within a single strain* (Wong and Good, 2022).

Regarding recombination, there is no doubt that linkage contributes to the patterns of genetic diversity we observe in the human gut as this topic remains an active area of research (e.g., Garud, Good, 2019; Roodgar et al., 2021; Good, 2022). However, the extent that an absence of recombination is necessary to preserve the ecological structure of strains within a human host is currently unknown, as published eco-evolutionary models of strain dynamics have primarily examined purely asexually reproducing populations. To reflect this point, in the revision I incorporated the reviewer's comment regarding the need to consider genetic linkage (line 29-31).

2c) "This confluence of ecological and evolutionary dynamics calls into question the feasibility of characterizing genetic diversity in the human gut." "Calls into question the feasibility" is a bit overstated. How about "requires new approaches and theory for

Author response: I have made the suggested edit (lines 32-33).

3) I am not swayed by the argument that there are always multiple strains in a subject (response to previous point #3). B fragilis is often found at very high abundances, and the presences of strain structure does not correlate with the abundance in that species, as would be expected if there was a detection limit problem. The author has not proved "a lack of genuine absences of strain structure" which is very strong claim that when not written in the double negative. This could be mitigated by putting a qualifier of "for most species" for most sentences in this paragraph.

Author response: The reviewer is correct that the implications of the gamma with regards to the existence of strains makes strong claims. In this manuscript I do not attempt to prove nor disprove this claim, I simply state the interpretation of the gamma based on past macroecological research performed at the species level (Grilli, 2020). In the revision I now clarify this point (lines 493-501). I have also incorporated the reviewer's suggestion and now include the qualifier "for several species" throughout the paragraph (lines 357, 495, 500, 505).

4) If I am understanding correctly, this paper is about the cross-host applicability of the SLM. However, the examples in the last paragraph of the discussion are all about within-host dynamics, which were investigated in a prior manuscript. This is confusing and perhaps misleading. Could the across-host applicability of the SLM help us understand how the

microbiome at a global scale might change in response to a global perturbation like global warming?

Author response: I apologize for the lack of clarity. The last paragraph was originally written at the request of a previous reviewer asking that I talk about the applicability of the SLM in general. To incorporate this comment and the comment of Reviewer 2, I have rewritten this paragraph to focus on the overall state of strain macroecology in-light of this study and how the assumption of stationarity could be leveraged to identify the effects of perturbations across-hosts (lines 567-587).

The reviewer does raise an interesting question regarding global perturbations. I would imagine that the temperature of endothermic systems like the human gut would be largely immune to slight (though globally important) gradual changes in the temperature of the climate. Scenarios where the gut ecology of a large number of human hosts are simultaneously perturbed would be ideal for evaluating across-host strain ecology. This scenario is most likely to occur in human experimental trials (e.g., effect of change in diet, novel drug testing, etc.). In this scenario the SLM could be used to examine statistical measures of over an ensemble of hosts (e.g., mean abundance over hosts of a given strain) relaxes over time towards a stationary state after a perturbation (i.e., $\langle x | t, x_0 \rangle$ => $\langle x \rangle$).

5) I understand that Figure 1 isn't about a goodness of fit, though the author has now done some analysis in this direction now anyway. I still don't really understand what I am supposed to learn from this data looking roughly gamma though. How do the lognormal and gamma distribution relate to the SFS expected for within-host evolution described in lead up to Figure 1? Can the author give an example of a generative process that wouldn't result in the patterns shown in Figure 1?

Author response: In my prior revision I fit a lognormal distribution at the reviewer's request that I include and compare an additional distribution as an alternative to the gamma. The purpose of Fig. 1c is to

1) Demonstrate that different species have similar distributions of within-host allele frequencies across hosts when rescaled and

2) Propose the gamma distribution as a potential model of allelic diversity *across* hosts.

This is the empirical motivation that leads to the SLM being identified as a reasonable model, as the stationary solution of the SLM predicts a gamma distribution and describes the temporal dynamics of strains within a single human host (Eqs. 10-12). In contrast, a Langevin equation that models linear ecological growth (as opposed to the logistic growth of the SLM) and environmental noise (the same form of noise as the SLM) predicts a lognormal distribution. This model

would be inappropriate since the lognormal did a comparatively poor job explaining the empirical distribution in Fig.1c relative to the gamma distribution.

However, different Langevin equations can produce the same probability distribution and it is worth considering whether such equations are viable alternatives to the SLM. In the manuscript I identified two Langevin equations of molecular evolution that also predict a gamma distribution (Eqs. 7-9; S1 Text). I describe the features of these models, their parameters, and how the assumptions necessary to explain the existence of genetic variants that are present at intermediate frequencies ($0 < f < 1$) over a large number of hosts are unrealistic (lines 409-439). As for a generative process that would *not* produce the pattern shown in Fig. 1c and would not produce a lognormal, an evolutionarily neutral allele *or* an allele on the background of an ecologically neutral strain would generate a Gaussian distribution over short timescales.

Finally, this section leads into an explicit test of whether the SLM as an ecological interpretation of the gamma is reasonable, where I find that the accuracy of the gamma is correlated with independent estimates of strain structure (Fig. 4), pointing to the SLM as a feasible ecological model. I have revised this section of the manuscript for clarity (Lines 367-386). I now also describe how distributions different from the gamma (i.e., lognormal or Gaussian) could emerge as a consequence of ecological or evolutionary dynamics (lines 373-385).

Reviewer #2

In their revision, the author worked to improve the readability of the manuscript, clarifying the concepts and the aim of the study. Overall, they did a good job. I thank the author for rewriting the manuscript to make it easier to follow. I still have some difficulties interpreting the equations, which is likely due to my unfamiliarity with the subject.

Author response: I thank the reviewer for their feedback.

Overall comments

Since the article was based on a previously published work (Wolff et al. 2023), I think it would improve understanding of the context of the study further if one or two lines summarizing the results of that study were added at line 69.

Author response: I have added the requested lines to the revision (lines 76-80).

While the last paragraph of the discussion addresses this, it is still not entirely clear to me how the model can be applied to dysbiosis. What are the implications of the prediction of the prevalence of a given gene in a fraction of hosts? What does that tell us about the microbiome?

Author response: I apologize to the reviewer for the lack of clarity. Much of this paragraph was written at the request of a past reviewer that asked for the macroecological implications of this work in terms of practical application to be discussed. To incorporate this comment and the comment of Reviewer 1, I have rewritten this paragraph to focus on the state of strain macroecology in-light of this study. Specifically, how the analyses in this study assume that strain dynamics are stationary with respect to time and how that assumption could be leveraged to determine when statistics calculated over the gut microbiomes of a large number of perturbed hosts approach stationarity (e.g., mean frequency or prevalence approaching a stationary value over time; lines 576-587).

References

Dapa, T., Wong, D. P., Vasquez, K. S., Xavier, K. B., Huang, K. C., & Good, B. H. (2023). Within-host evolution of the gut microbiome. *Current Opinion in Microbiology*, *71*, 102258. https://doi.org/10.1016/j.mib.2022.102258

Garud, N. R., Good, B. H., Hallatschek, O., & Pollard, K. S. (2019). Evolutionary dynamics of bacteria in the gut microbiome within and across hosts. *PLOS Biology*, *17*(1), e3000102. https://doi.org/10.1371/journal.pbio.3000102

Good, B. H. (2022). Linkage disequilibrium between rare mutations. *Genetics*, *220*(4), iyac004. https://doi.org/10.1093/genetics/iyac004

Good, B. H., Martis, S., & Hallatschek, O. (2018). Adaptation limits ecological diversification and promotes ecological tinkering during the competition for substitutable resources. *Proceedings of the National Academy of Sciences*, *115*(44), E10407–E10416. https://doi.org/10.1073/pnas.1807530115

Good, B. H., & Hallatschek, O. (2018). Effective models and the search for quantitative principles in microbial evolution. *Current Opinion in Microbiology*, *45*, 203–212. https://doi.org/10.1016/j.mib.2018.11.005

Grilli, J. (2020). Macroecological laws describe variation and diversity in microbial

communities. *Nature Communications*, *11*(1), 4743. https://doi.org/10.1038/s41467-020-18529-y

Roodgar, M., Good, B. H., Garud, N. R., Martis, S., Avula, M., Zhou, W., Lancaster, S. M., Lee, H., Babveyh, A., Nesamoney, S., Pollard, K. S., & Snyder, M. P. (2021). Longitudinal linked-read sequencing reveals ecological and evolutionary responses of a human gut microbiome during antibiotic treatment. *Genome Research*, *31*(8), 1433–1446. https://doi.org/10.1101/gr.265058.120

Shoemaker, W. R., Chen, D., & Garud, N. R. (2021). Comparative Population Genetics in the Human Gut Microbiome. *Genome Biology and Evolution*, *evab116*. https://doi.org/10.1093/gbe/evab116

Wolff, R., Shoemaker, W., & Garud, N. (2023). Ecological Stability Emerges at the Level of Strains in the Human Gut Microbiome. *MBio*, *0*(0), e02502-22.

Wong, D., & Good, B. (2022). Ecological diversification of rapidly adapting populations. In APS March Meeting Abstracts (Vol. 2022, pp. M05-011).
