# Supplementary material for: A macroecological perspective on genetic diversity in the human gut microbiome
Source: PLoS One. 2023 Jul 21;18(7):e0288926. doi: 10.1371/journal.pone.0288926 (PMC10361512; doi:10.1371/journal.pone.0288926)
Supplement: Attachment — Submitted filename: 20230603_1112_point_by_point.pdf [file pone.0288926.s017.pdf]

Editor Comments

Thank you for submitting your manuscript to PLOS ONE. The manuscript is now almost ready for acceptance. However, we have a new reviewer, who has a minor comment, which the author could potentially comment on, and further improve the manuscript.

> **Author response: I thank the reviewer for their feedback. Below I address Reviewer #3's comment.**

Reviewer #1

No additional comments.

Reviewer #2

No additional comments.

Reviewer #3

The author has addressed all prior reviewer concerns. I have no further concerns, and I commend the author on an interesting piece. One minor point that the author may want to consider is to discuss de novo evolution of coexisting strains from a recent ancestor within the lifespan of a human host. Most of the discussion of ecological dynamics of strains appears to assume rather distant lineages (separated by > thousands of SNPs) colonizing the same host. However, the Zhao et al. reference (ref 1) shows two B. fragilis strains within an individual, separated by a handful of SNPs, where both lineages coexist for a year or more (i.e., stationary dynamics - one lineage does not sweep the other). This would suggest a kind of sympatric speciation (transition from the evolutionary to the ecological regime) is possible over very short timescales.

> **Author response: The reviewer raises an interesting question. They are correct in their assessment that I implicitly assumed that strains are diverged by many SNVs. This is due to the limitations of algorithms such as strain-finder, which uses the distribution of allele frequencies to test for the existence of strain structure within a host, where strain structure cannot be determined if the strains within a host are genetically diverged at only a handful of sites.**
>
> **However, strains must ultimately have descended from a common ancestor, and it is worth discussing whether the existence of recently evolved strains can impact the patterns evaluated in this study. A newly evolved strain within a single host is analogous to a species that is only present in a single host. Given that the sampling form of the gamma distribution used in this study succeeds at predicting the prevalence of these species (Grilli, 2020), it should, in principle, be capable of predicting the prevalence of a SNV observed in a single host due to recently evolved**

strain structure. Across species we find that predictions for low prevalence alleles consistently fail though they tend to succeed for high prevalence alleles (e.g., Figs. S5). It is reasonable to interpret the lack of predictive success for low prevalence alleles as a consequence of said allele being present in a low number of hosts due to evolutionary dynamics, rather than its presence being a reflection of ecological strain structure. However, this interpretation does not mean that recently diverged strains are absent in the cohort of human hosts used in this study. Rather, it is possible that the macroecological lens applied here is insensitive to recently diverged strains that have colonized a low number of hosts similar to the number of hosts that we would expect to find a given allele present at intermediate frequencies due to evolutionary dynamics (e.g., repeated mutation).

In the revised manuscript I now summarize the above response to address the reviewer's comment (lines 574-595).

References

Grilli, J. (2020). Macroecological laws describe variation and diversity in microbial communities. *Nature Communications*, *11*(1), 4743. https://doi.org/10.1038/s41467-020-18529-y
